# High microbial diversity, functional redundancy, and prophage enrichment support the success of the yellow pencil coral, *Madracis mirabilis,* in Curaçao’s coral reefs

**DOI:** 10.1128/msystems.01208-25

**Published:** 2025-10-16

**Authors:** Bailey A. Wallace, Natascha S. Varona, Alexandra K. Stiffler, Mark J. A. Vermeij, Cynthia Silveira

**Affiliations:** 1 Department of Biology, University of Miami 124503, Coral Gables, Florida, USA; 2 Department of Freshwater and Marine Ecology, Institute for Biodiversity and Ecosystem Dynamics, University of Amsterdam 100598, Amsterdam, the Netherlands; 3 Carmabi Foundation, Piscaderabaai z/n, Willemstad, Curaçao; 4 Department of Marine Biology and Ecology, Rosenstiel School of Marine, Atmospheric, and Earth Science, University of Miami 551192, Miami, Florida, USA; Quadram Institute Bioscience, Norwich, Norfolk, United Kingdom

**Keywords:** bacteriophage, prophage, metagenome-assembled genome, coral resilience, bacteria

## Abstract

Coral reefs have undergone extensive coral loss and shifts in community composition worldwide. Despite this, some coral species appear naturally more resistant, such as *Madracis mirabilis* (herein *Madracis*). *Madracis* has emerged as the dominant hard coral in Curaçao, comprising 26% of coral cover in reefs that declined by 78% between 1973 and 2015. Although life history traits and competitive mechanisms contribute to *Madracis*’s success, these factors alone may not fully explain it, as other species with similar traits have not shown comparable success. Here, we investigated the potential role of microbial communities in the success of *Madracis* on Curaçao reefs by leveraging a low-bias bacterial and viral enrichment method for metagenomic sequencing of coral samples, resulting in 77 unique bacterial metagenome-assembled genomes and 2,820 viral genomic sequences. Our analyses showed that *Madracis*-associated bacterial and viral communities are 12% and 20% richer than the communities of five sympatric coral species combined. The *Madracis*-associated bacterial community was dominated by *Ruegeria* and *Sphingomonas*, genera that have previously been associated with coral health, defense against pathogens, and bioremediation. These communities also displayed higher functional redundancy, which is often associated with ecological resilience. The viral community exhibited a 50% enrichment of proviruses relative to other corals. These proviruses had the genomic capacity to laterally transfer genes involved in antibiotic resistance, central metabolism, and oxidative stress responses, potentially enhancing the adaptive capacity of the *Madracis* microbiome and contributing to *Madracis*’s success on Curaçao’s reefs.

**IMPORTANCE:**

Understanding why some coral species persist and thrive while most are in fast decline is critical. *Madracis mirabilis* is increasingly dominant on degraded reefs in Curaçao, yet the role of microbial communities in its success remains underexplored. This study highlights the potential role of *Madracis*-associated bacterial and viral communities in supporting coral resilience and competitive success. By identifying key microbial partners and viral genes that may enhance host stress tolerance and defense against pathogens, we broaden the understanding of how the coral holobiont contributes to species persistence under environmental stress. These insights are valuable for predicting key microbial community players in reef interactions and may inform microbiome-based strategies to support coral conservation and restoration.

## INTRODUCTION

A promising approach to coral reef conservation and restoration is the identification and exploration of coral species and reefs that are doing better than expected, given the pressures they are under (1). These interesting cases may provide insights into the physiological, genetic, and microbial adaptations that enable survival and stability under environmental stressors like warming, acidification, or reduced water quality. In the Caribbean, *Madracis mirabilis*, commonly known as yellow pencil coral, exemplifies this resilience despite dramatic losses in coral cover throughout the region. (We acknowledge the proposed renaming of *Madracis mirabilis* to *Madracis auretenra*. However, due to ongoing concerns over the validity of the type specimen used in the revision by Locke et al. [2] and the widespread and longstanding use of *M. mirabilis* in the literature, we continue to use *M. mirabilis* in this article for clarity and consistency.) The coral reefs of Curaçao are no exception when it comes to the global trends of reef decline over the past few decades (3). Anthropogenic stressors have contributed to reduced coral cover, elevated algal presence, and shifts in community composition (4). Despite a 78% decline in hard coral coverage from 1973 to 2015 in Curaçao, *M. mirabilis* (*Madracis*) has become the dominant hard coral species, accounting for 26% of coral cover and even increasing in some shallow reef sites (5–7).

Across Curaçao’s reef, *Madracis* experiences the highest growth and percent cover in areas proximal to chronic anthropogenic stressors, including nutrient loading, pollution, and organic matter inputs (7, 8). Typically, these stressors lead to community-level shifts and increasingly microbialized reefs, which are detrimental to coral health (9, 10). Microbialization is stimulated by organic matter inputs, resulting in the proliferation of copiotrophic bacteria, which contribute to the formation of hypoxic zones through elevated heterotrophic respiration and often include opportunistic coral pathogens (10). Despite these challenges, *Madracis* demonstrates remarkable resilience, showing relative insensitivity to coral diseases that affect many other species. Life history traits, such as fast growth rates and high population turnover, make weedy species of coral, like *Madracis*, particularly resilient to the impacts of habitat degradation (11). As an efficient heterotroph, *Madracis* consumes zooplankton, bacteria, and particulate matter from the water column, which may offset the typical decreases in autotrophic feeding (i.e., photosynthesis) caused by environmental stressors (12–14). *Madracis* not only thrives under environmental and microbial stress but also coexists with a dense community of benthic competitors. Its unique morphology, where tissue at the base of each branch recedes as the colony expands, creates space for diverse communities of crustose coralline algae, sponges, algal turfs, cyanobacterial mats, and other benthic organisms. This interactions with competitors may explain its heightened aggression through mesenterial filaments and sweeper tentacles (8). While these life history, nutrition, and aggression traits likely contribute substantially to *Madracis*’s expansion and dominance in Curaçao, numerous coral species share similar characteristics without achieving comparable success (6, 7, 15). Thus, these traits may not fully explain *Madracis*’s disproportionate success.

Microorganisms of the coral holobiont are both crucial to coral fitness and highly sensitive to the physiological status of their host (16). The composition of microbial communities within the coral holobiont is shaped by the dynamic interactions between these symbiotic microorganisms and environmental conditions, encompassing both external factors (e.g., temperature, nutrient availability, light availability) and internal host-specific factors (e.g., genotype, immune status, metabolic needs) (17–19). Members of the microbiome may also be vertically transmitted, further contributing to host-specific community structures (20). Within this framework, a beneficial community is selected in response to multiple factors, including the host and its environmental and physiological context (19). Dysbiosis in coral-associated microbial communities has been identified as a key contributor to disease (21, 22), with resident bacteria acting directly as opportunistic pathogens under certain environmental pressures (23–27). Other microbiome members provide significant benefits to their coral hosts, as highlighted by the Beneficial Microorganisms for Corals concept (18, 28). Beneficial microbes are thought to outcompete harmful microbes and even reverse dysbiosis within the coral holobiont through key functions like pathogen control, neutralization of toxic compounds, and nutrient cycling (29–33). Though microbial community composition can differ widely across coral species, flexibility in microbial partnerships may provide advantages to corals on short timescales, in contrast to genetic mutation and natural selection (19). The ability of microbes to act both as pathogens and as agents of adaptability highlights the complex dynamics of coral-microbe relationships and their potential to determine competitive success.

Viruses may also play a significant role in modulating microbial dynamics within corals. Bacteriophages, viruses that infect bacteria, act as agents of horizontal gene transfer and exert top-down control on bacterial communities (34–36). In other systems, viral predation can determine the colonization success of invading bacterial strains (37) and function as a form of mucosal immunity (36, 38, 39). Viruses can also carry auxiliary genes that can modulate host metabolisms, virulence, and other phenotypes (35, 40). Long-term relationships between viruses and bacteria through viral genome integration (forming a prophage) can provide the bacterium with several advantages, such as immunity to superinfection by related phages and the acquisition of novel genes, which can spread beneficial traits like antibiotic resistance or virulence factors (35). Moreover, prophage persistence can help maintain genetic diversity within bacterial populations, promoting evolutionary flexibility and adaptation in fluctuating environments (41, 42).

Here, we describe the bacterial and viral communities associated with *Madracis mirabilis*, the corals it interacts with, and seawater from the coral boundary layer to uncover the potential roles of these microbes in *Madracis*’s ecological success. We hypothesize that the *Madracis* microbiome displays unique traits related to community diversity and genetic makeup that can contribute to supporting this coral’s persistence.

## MATERIALS AND METHODS

### Sample collection

**Fig 1:**
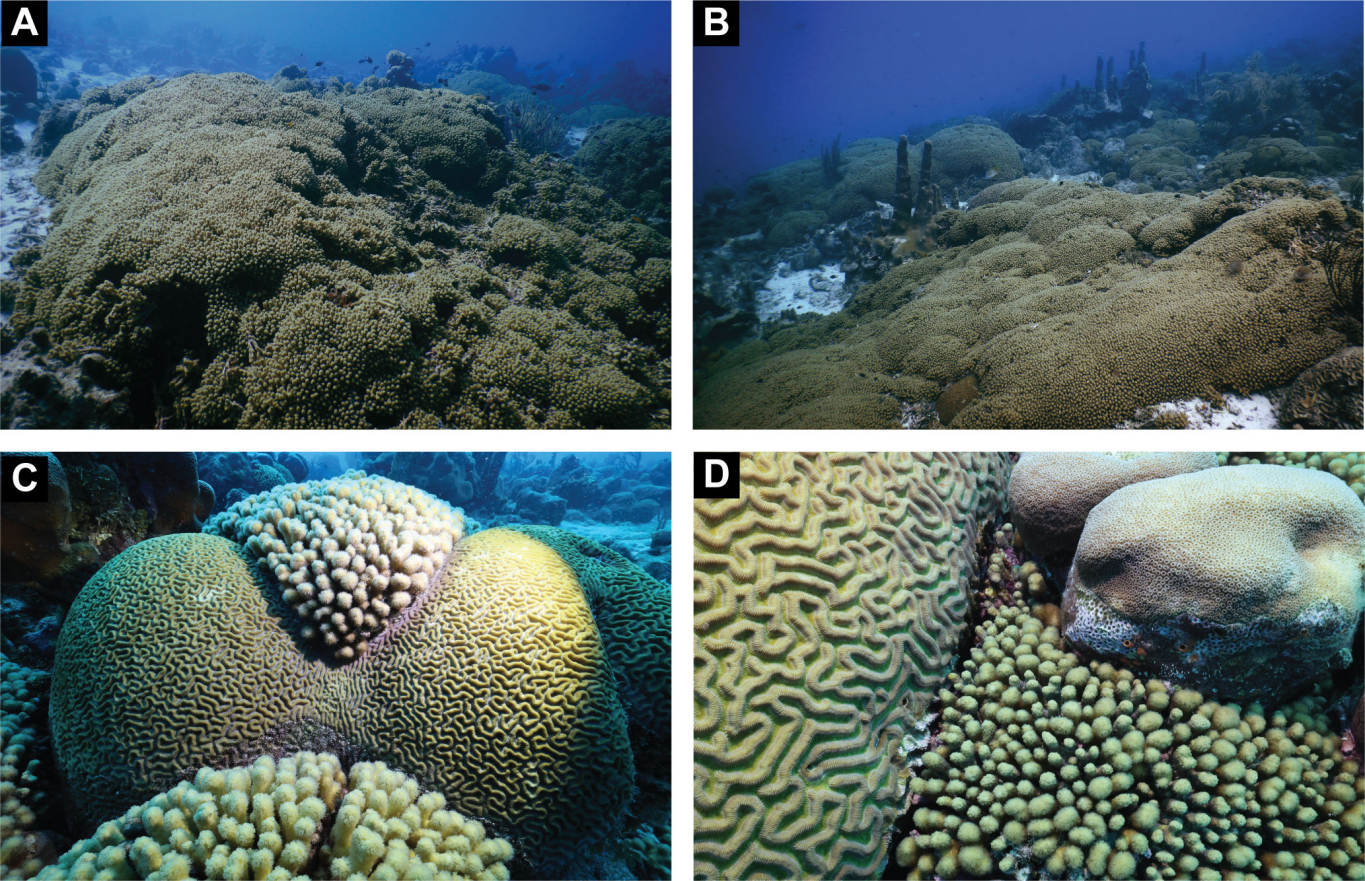
*Madracis* colonies in the reefscape and their interactions with other benthic organisms. (**A, B**) *Madracis* colonies are widely distributed across the reefscape, contributing significantly to coral cover. (**C, D**) *Madracis* colonies interacting with neighboring coral species, highlighting competition for space.

Images, coral biopsies, and coral boundary layer (CBL) seawater samples were collected at 12 sites along the southwestern coast of Curaçao in June of 2022 (Fig. S1A; Table S1). At the time of sampling, *Madracis* was the dominant scleractinian coral across surveyed reef sites on Curaçao, visibly dominating the benthic reefscape (Fig. 1A and B). *Madracis* colonies were frequently observed in direct competition with other benthic organisms, including neighboring coral species (Fig. 1C and D). At each site, a *Madracis* patch interacting with one of five other coral species was sampled, as shown in Fig. S1B. Interactions were imaged at high resolution for quantification of interaction zone outcomes (*N* = 11 with one site lost; see Supplemental methods for details). Coral biopsies of approximately 1 cm^3^ (containing mucus, tissue, and skeleton) were collected using a chisel and hammer and placed in Ziplock polyethylene bags with ambient seawater. Samples were placed on ice during transfer to the laboratory at the CARMABI research station (maximum 1 h transfer time), where ambient seawater was removed, and coral samples were flash-frozen and stored at −80°C until later processing. For comparison with the microbiome in the surrounding environment, CBL seawater samples were collected from four areas around each *Madracis* patch using a 500 mL syringe equipped with a 30 cm long flexible tube: within the *Madracis* fingers (inter-branch; IB1), the boundary layer overlaying the *Madracis* (within 10 cm of the coral surface; BL1), the boundary layer over the interaction zone (BL2) and the boundary layer of an upstream coral of the same interacting species (BL3). The BL3 sample provides a species-specific comparison that would account for the background microbiome signal of that coral species in the absence of a direct interaction with *Madracis*. This design allowed us to better isolate microbial features that may be associated with the interaction itself (Fig. S1B).

### Epifluorescence microscopy of coral boundary layer seawater

For epifluorescence microscopy, 1 mL of raw CBL sample was fixed with paraformaldehyde (2% final concentration; Thermo Scientific Chemicals, Waltham, MA, USA) and vacuum-filtered onto a 0.02 µm Anodisc (Cytiva, Marlborough, MA, USA). Anodiscs were air-dried, stored flat at −20°C, and transported to the University of Miami on ice. In the laboratory, nucleic acid-containing particles on the filters were stained by placing the filter on a 100 µL drop of SYBR Gold Nucleic Acid Gel Stain (10× final concentration; Invitrogen, Waltham, MA, USA) for 10 min. Excess stain was removed by wetting the Anodisc from below with two drops of 100 µL molecular-grade water and gently blotting the bottom of the filter with a lint-free wipe. Filters were air-dried for 30 min without exposure to light and mounted between a glass microscope slide and coverslip with 20 µL of mounting solution (ascorbic acid [0.1%], phosphate-buffered saline [1×], and 0.02 µm-filtered glycerol [50%]). Slides were visualized using oil immersion at 630× magnification (63× objective and 10× eyepiece) on a ZEISS Axio Imager.A2 using ZEN image processing software (ZEISS, Oberkochen, BW, GER). For each slide, 10 images for BL1, BL2, and BL3, and five images for IB1 were captured as technical replicates using an Axiocam 506 mono camera. The mean number of cells and virus-like particles (VLPs) across these fields of view was scaled to the total area of the Anodisc, yielding the count of cells and VLPs per mL in each CBL sample. Two sites, “Kokomo Beach” and “Tugboat Beach,” were removed from the analysis due to low-quality slides, which affected their reliability for downstream analyses.

### DNA extractions and sequencing

For the CBL seawater metagenomes, four 500 mL samples from each site were 8.0 µm-filtered (polycarbonate track-etched membrane filter; Whatman, Buckinghamshire, UK) and precipitated overnight with 10% polyethylene glycol 8000 (PEG; Fisher BioReagents, Pittsburgh, PA, USA). The next morning, PEG-precipitated bacteria and viruses were collected on a 0.22 µm Sterivex PES filter (MilliporeSigma, Burlington, MA, USA) and frozen at −20°C for later processing. DNA from Sterivex filters containing CBL seawater samples from six sites (*N* = 21) was extracted using a modified Nucleospin Tissue Kit protocol (Macherey-Nagel, Düren Nordrhein-Westfalen, Germany) (43). Briefly, the Sterivex filters were first thawed and air-dried by forcing air through with a syringe. A pre-lysis step was performed by capping one end of the filter and adding 360 µL of Buffer T1 and 50 µL of proteinase K directly to the Sterivex, followed by overnight incubation at 56°C on a rotating spit. The next day, 400 µL of buffer B3 was added, and the samples were incubated at 70°C for 30 min. Lysates were extracted from the Sterivex filters using a luer lock syringe and transferred to 2 mL tubes. The procedure continued from step 4 of the NucleoSpin Tissue kit protocol for human or animal tissues and cultured cells. Seawater samples served as environmental controls, providing background context for microbial and viral communities present in the surrounding water column. These samples allow for differentiation between host-associated and free-living taxa. DNA libraries were prepared using the Nextera XT DNA Library Preparation Kit (Illumina) and IDT Unique Dual Indexes using 1 ng of DNA. Fragmentation was performed with Illumina Nextera XT fragmentation enzyme, followed by PCR amplification (12 cycles) and purification with AMpure magnetic Beads (Beckman Coulter). Libraries were quantified using a Qubit 4 fluorometer (ThermoFisher Scientific, Waltham, MA, USA) and a Qubit dsDNA HS Assay Kit (Invitrogen, Waltham, MA, USA). Libraries were sequenced on a NovaSeq platform 2 × 150 bp (Illumina, San Diego, CA, USA).

DNA extractions from 24 coral samples followed a host depletion protocol based on tissue disruption, DNase treatment, and size fractionation (44, 45). To preserve both microbial and viral communities in a single metagenomic sample, we did not physically separate viruses and cells prior to DNA extraction. This approach avoids biases introduced by chloroform treatment or CsCl gradients and allows for the simultaneous assembly of bacterial and viral genomes from the same metagenomic sample (45). Because our approach does not purify free viral particles, and coral tissues are densely colonized by microbes, these samples have much higher biomass than viromes generated from viral purification protocols and do not require amplification for shotgun sequencing. Thus, additional amplification biases are avoided (46), reducing concerns surrounding negative controls for low-biomass sample amplification via PCR before sequencing (47, 48). Nevertheless, negative buffer controls (*N* = 3) were included by processing sterile artificial seawater through the full extraction protocol, from sampling vials, processing with a mortar and pestle, and extraction with the same kit. No detectable DNA was recovered, and therefore, no sequencing or bioinformatic batch correction was performed in subsequent steps.

Genomic DNA was quantified using the Qubit 2.0 Fluorometer (ThermoFisher Scientific, Waltham, MA, USA), and libraries were prepared with the NEBNext Ultra II DNA Library Prep Kit for Illumina following the manufacturer’s recommendations. Genomic DNA was fragmented by acoustic shearing (Covaris S220), cleaned, end-repaired, and adapter-ligated, followed by PCR enrichment. Libraries were validated using a High Sensitivity D1000 ScreenTape on the Agilent TapeStation (Agilent Technologies, Palo Alto, CA, USA) and quantified by real-time PCR (Applied Biosystems, Carlsbad, CA, USA). Libraries were sequenced on an Illumina NovaSeq X Plus instrument 2 × 150 bp. *Orbicella faveolata* samples (*N* = 19) collected in Curaçao in 2021 and extracted and sequenced with the same methods were also incorporated into this data set, expanding the total number of sampling sites by three (Fig. S1A). The collection and sequencing details of the 2021 samples were previously reported (45). Following sequencing, we observed variation in sequencing depth across coral samples, with several libraries yielding comparatively low read counts (Table S1). These differences in sequencing effort were considered in downstream diversity analyses, and collector’s curves were used to assess the effect of sequencing depth on diversity metrics (Fig. S2).

### Identification of bacterial and viral sequences

Raw metagenomic reads were adapter-trimmed and quality-filtered (trimq = 30, maq = 30) with BBDuk v39.01 (49), generating over 900 M quality-controlled, paired-end reads as quantified by FastQC (50). For bacterial community analysis, metagenomic reads were analyzed for taxonomic classification using Kaiju v1.10.1 with the proGenomes Database v3 (51, 52). The relative abundances of bacterial genera were calculated using the following equation:


$$\text{relative abundance of bacterial genus}=\frac{r(genus)}{r(bacteria)}\times 100\%$$

where *r*(genus) is the total number of reads matched to a particular bacterial genus and *r*(bacteria) is the total number of reads matched to the domain Bacteria. Bacterial metagenome-assembled genomes (bMAGs) were generated by combining and improving the single-sample coverage binning outputs from MaxBin2 v2.2.7 (53), MetaBat2 v2.15 (54), and CONCOCT v1.1.0 (55) with metaWRAP v1.2.1 (56). Bins with ≥50% completion and ≤10% contamination (*N* = 2,112) were classified with GTDB-Tk v2.4.0 (GTDB release 220) (57). The anvi-compute-genome-similarity and anvi-dereplicate-genomes modules of Anvi’o v8 (58) were used to dereplicate bMAGs at a similarity threshold of 95%, generating 77 species-level representative bMAGs. To identify proviruses within these genomes, VIBRANT v1.2.1 (59) and geNomad v1.7.4 (60) were run separately on the bMAGs. A phylogenetic tree of the representative bMAGs was constructed using the GTDB-Tk infer module. For viral community analysis, quality-controlled reads from each sample were assembled with metaSPAdes v3.15.5 using default parameters (61). The resulting assemblies were analyzed using the ViWrap v1.3.1 pipeline (62), utilizing both VIBRANT and geNomad viral identification tools. Within this pipeline, viral contigs were binned using VRhyme v1.1.0 (63) and taxonomically classified using the NCBI RefSeq viral protein, VOG HMM, and IMG/VR v4 databases (64–66). All resulting binned and unbinned viral genomes and genome fragments identified with the two distinct viral identification pipelines were pooled and dereplicated at MIUViG standards (95% average nucleotide identity [ANI] over 80% alignment fraction [AF]) with CheckV’s (v1.0.1) rapid genome clustering based on pairwise ANI (67), resulting in 13,113 unique viral sequences. These viruses were further quality-filtered by removing all sequences with no viral genes and CheckV quality “Not-determined,” resulting in a final database of 2,820 viruses. Reads from each sample were mapped at 95% identity to the de-replicated viral and bMAG databases with SMALT v0.7.6, and relative fractional abundances of viral sequences, bacterial contigs, and bMAGs were calculated using the following equation:


$$f\left(i\right)=\frac{r\left(i\right)}{T\left(j\right)}\times \frac{L\left(mean\right)}{L\left(i\right)}.$$

Here, *r*(*i*) is the number of reads mapped to each viral or bacterial genome, *T*(*j*) is the total number of reads mapped in a given sample, *L*(mean) is the mean genome length in the data set, and *L*(*i*) is the length of the mapped sequence (68). Only sequences with >10 reads mapped were considered present in a sample. For bMAG abundances, we calculated the average relative fractional abundance of each bMAG across each group by dividing the fractional abundance of a bMAG in a given sample by the sum of all bMAG fractional abundances in that sample and averaging across groups. This method controls for variation in sequencing depth, genome length, and differences in the number of bMAGs recovered per sample (68). Genome annotation of viruses and bMAGs was performed with MetaCerberus v1.4.0 (69). The abundances of functional gene categories encoded by proviruses, bMAGs, and bacterial contigs were calculated based on the relative fractional abundance of their host genomes, which were summed within each sample, and then averaged across the coral groups (*Madracis* and others). Best hits were further filtered by database to select the 15 most differentially abundant gene pathways, clusters of orthologous genes (COG) (all available pathways), and Kyoto Encyclopedia of Genes and Genomes (KEGG) (filtered for pathways containing the keyword “metabolism”), between *Madracis* and other corals. To test for statistical significance among the most differentially abundant functional categories between *Madracis* and other corals, we performed Wilcoxon rank-sum tests. *P* values were adjusted using the Benjamini-Hochberg method to control the false discovery rate. For bMAG symbiosis gene identification, MetaCerberus gene products were filtered using keywords to identify genes previously identified as relevant to bacterial-host symbioses (70). *Madracis* indicator viruses (described below) encoding integrases (*N* = 3) and two high-quality genomes of *Caudoviricetes* proviruses identified by CheckV were visualized with genoPlotR (71). Viral host prediction was performed with iPHoP v1.3.3 for the indicator viruses and SIMPER viruses (72), and for the annotated CheckV-identified proviruses, hosts were predicted using BLASTn searches of their bacterial flanking regions.

### Statistical analysis

Differences between groups in the microscopy-based abundance data were tested with an analysis of variance (ANOVA), followed by Tukey HSD tests to identify significant pairwise differences. To assess the similarity between viral and microbial community profiles, the Bray-Curtis similarity algorithm was applied to bacterial genus relative abundances and viral relative fractional abundances (vegan: vegdist) and visualized with non-metric multidimensional scaling (NMDS) (vegan: metaMDS). Significant differences between groups were identified using Adonis permutational ANOVAs (adonis2; 999 permutations) and pairwise Adonis tests (pmartinezarbizu: pairwise.adonis2). The homogeneity of multivariate dispersion was evaluated on the Bray-Curtis distance matrices (vegan: betadisper). Significant differences in NMDS ordination of bacterial and viral communities were observed when grouped by sample type (coral vs CBL seawater) and year (2021 vs 2022), yet these differences may also be due to the coral species (Fig. S3; Tables S2 to S4).

To describe which bacterial genera and viruses contributed to the between-group differences identified by the permutational multivariate analysis of variance tests, we used similarity percentages (vegan: SIMPER) analysis. Only the 20 bacterial genera and viral sequences with the highest contribution to the dissimilarity between coral groups were reported. To identify which viral community members had high specificity and fidelity to the *Madracis* community, indicator species analyses were used (indicspecies: multipatt). Diversity metrics, including Shannon diversity, Simpson diversity, Evenness, and Richness, were calculated for the bacterial genera, viral genomes, and the functional and metabolic pathway annotations of bacterial contigs in each coral group (vegan: diversity). Functional redundancy (${FR}_{\alpha }$) within the bacterial communities was derived from the functional annotations of bacterial contigs and calculated as


$$FR_{\alpha }=TD_{\alpha }-FD_{\alpha }$$

where ${TD}_{\alpha }$ is the alpha taxonomic diversity at the genus level (Simpson index; SYNCSA v1.3.4:rao.diversity) and ${FD}_{\alpha }$ represents the mean functional distance between any two randomly chosen genera in the community (Rao’s quadratic entropy; SYNCSA v1.3.4:rao.diversity) (73). To test the effects of seven coral samples that yielded significantly fewer sequencing reads (<1 M reads) and *Orbicella faveollata* (OFAV) samples collected in a different year (with different beta-dispersion in bacterial [*P* = 0.0011, Tukey HSD] and viral [*P* = 0.0005, Tukey HSD] communities), we removed those samples and reanalyzed the data set, finding largely similar results (Tables S5 to S8). However, we note that bacterial alpha diversity metrics were sensitive to the removal of low-depth samples. Shannon diversity became significantly different between *Madracis* and other corals after removal, while the difference in richness was no longer significant (Table S7).

## RESULTS

### Bacterial and viral communities in coral boundary layer seawater

**Fig 2:**
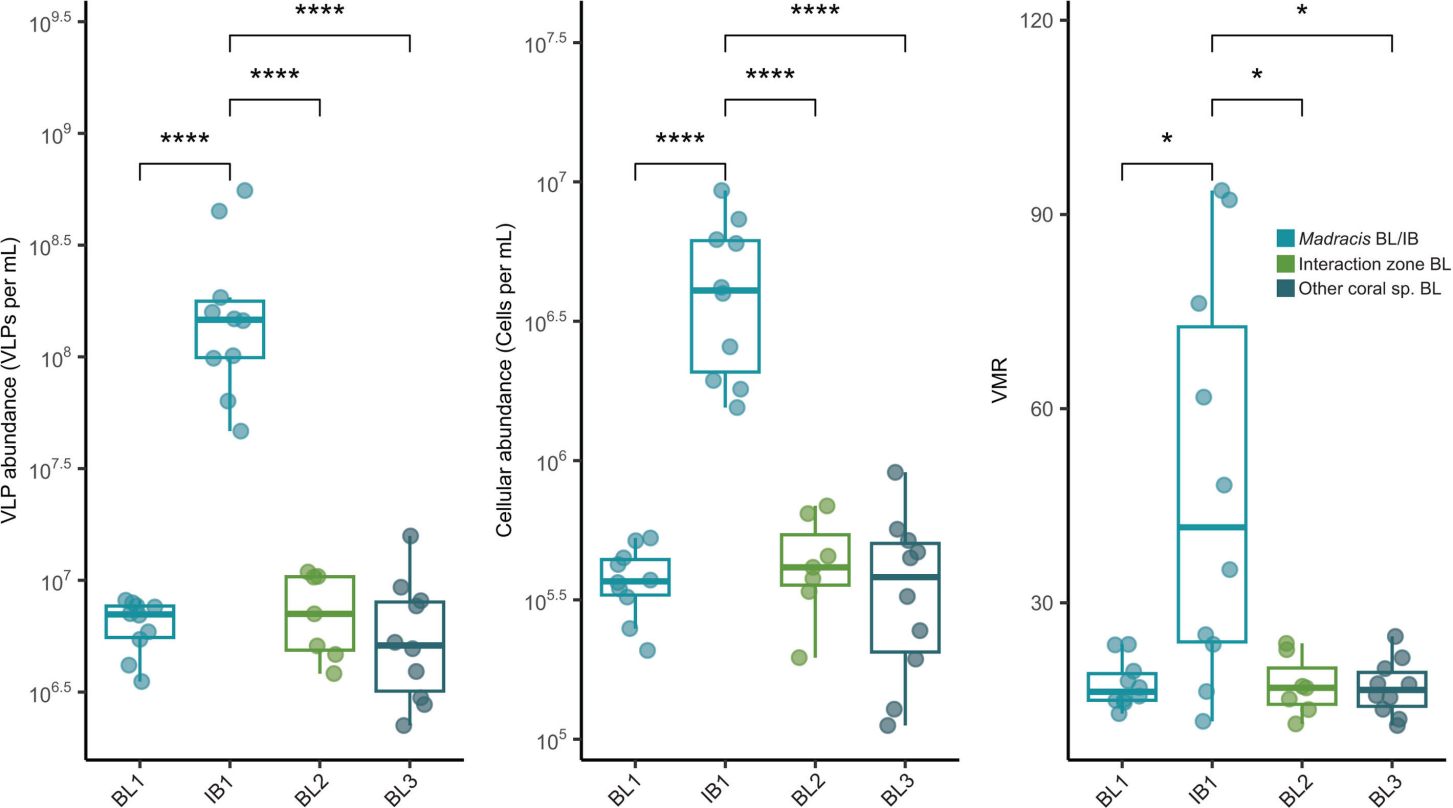
Microbial abundances in the coral boundary layer and interbranch seawater. Epifluorescence microscopy counts of virus-like particles (VLPs), cells, and virus-to-microbe ratios (VMR) for seawater samples spanning *Madracis* interactions. Mean counts per mL of sample and VMRs are displayed for each of the four seawater sample types, including the *Madracis* boundary layer (BL1), *Madracis* interbranch (IB1), interaction zone boundary layer (BL2), and other coral species boundary layer (BL3). Only statistically significant differences are displayed (significance codes: 0.0001, ****; 0.001, ***; 0.01, **; and 0.05, *).

Interbranch seawater from between the *Madracis* fingers (IB1; *N* = 10) had significantly higher viral abundance [ANOVA: F(3, 33) = 11.03, *P* = 3.59e−05], bacterial abundance [F(3, 33) = 20.99, *P* = 8.62e−08], and virus-to-microbe ratios [VMR; F(3, 33) = 8.65, *P* = 0.00023] compared to all boundary layer samples collected above the corals (BL1, *N* = 10; BL2, *N* = 7; and BL3, *N* = 10; Fig. 2; Table S9). Viral and bacterial abundances in IB1 were 26- to 31-fold and 10- to 12-fold higher, respectively, while VMRs were threefold higher than in BL samples. No significant differences were detected among BL1, BL2, and BL3 (Table S10). Despite differences in cellular and viral abundances between the interbranch and boundary layer samples, bacterial community composition only differed between the interbranch samples (IB1) and the boundary layer overlaying the *Madracis* [BL1; F(1, 9) = 3.42, *P* = 0.025; Table S11]. Viral communities were also similar among boundary layer (BL) samples but differed significantly between IB1 and both BL1 [F(1, 9) = 4.27, *P* = 0.003] and BL2 [F(1, 8) = 1.87, *P* = 0.017]. Combined boundary layer and interbranch communities were distinct from those associated with coral tissue (Tables S3 and S4). Given the similarity among boundary layer communities, we combined all seawater samples into a single coral boundary layer category, termed “CBL seawater,” for downstream comparisons.

### Interaction outcomes

To assess whether bacterial and viral communities influenced the outcomes of direct competition between *Madracis* and other coral species, we quantified these interactions according to Fig. S4A. *Madracis*’s interaction outcomes were generally not species specific, but other coral species lost more against *Madracis* (Fig. S4B) than against other benthic substrates (Fig. S4C). To test for cross-species differences associated with interaction outcomes, we grouped all interacting coral samples (CRL3 and CRL5) by competitive outcome (win vs lose), regardless of species identity. Bacterial and viral communities were not significantly different in winning or losing interaction outcomes [F(1, 22) = 0.896, *P* = 0.511 for bacteria and F(1, 22) = 1.571, *P* = 0.139 for viruses; Fig. S5]. Ratios of *Bacteroidetes* to *Bacillota* (previously known as *Firmicutes*), which are markers of dysbiosis (74–77), were similar between winning and losing corals (Fig. S6). To investigate whether low-abundance or other key taxa were associated with these competitive outcomes, we additionally performed an indicator species analysis. While no bacterial genera were significantly associated with winning corals, seven genera were significantly associated with losing corals. Although these taxa lacked complete specificity or fidelity to losing samples, their consistent enrichment suggests they may represent microbial indicators of competitive stress or compromised holobiont condition (Table S12). Due to the lack of strong differences between winning and losing corals in these analyses, we did not pursue this line of inquiry in subsequent analyses.

### Bacterial communities associated with *Madracis*

**Fig 3:**
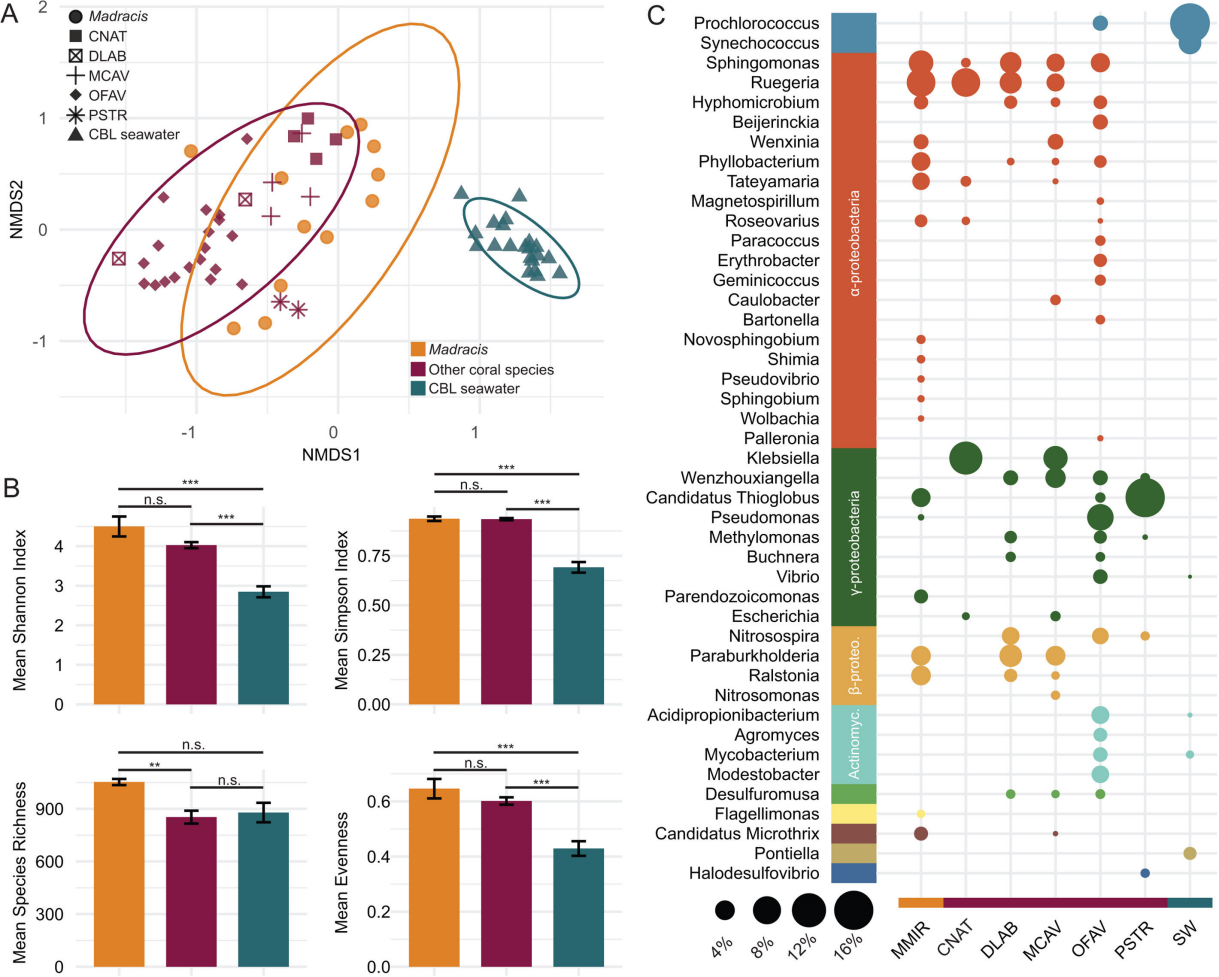
Bacterial community dissimilarity between *Madracis*, other coral species, and coral boundary layer (CBL) seawater. (**A**) Non-metric multidimensional scaling (NMDS) plot based on Bray-Curtis distances calculated from the relative abundance at the genus level with 999 permutations. Ellipses denote a 95% confidence interval for the sample types. (**B**) Mean diversity indices of bacterial genera, including Shannon index, simpson index, evenness, and richness. Significance codes: 0, ***, and 0.001, **. (**C**) Relative abundances of bacterial genera by coral species/seawater for genera representing >1% of the bacterial community. The size of the circles indicates relative abundance (%), and the color of the bubbles indicates bacterial classes. Coral species names are abbreviated as follows: *Colpophyllia natans* (CNAT), *Diploria labyrinthiformis* (DLAB), *Montastraea cavernosa* (MCAV), *Madracis mirabilis* (MMIR), *Orbicella faveolata* (OFAV), *Meandrina meandrites* (MMEA), and *Pseudodiploria strigosa* (PSTR).

Bacterial community assemblages differed among *Madracis*, other coral species, and the combined CBL seawater [F(2, 61) = 28.184, *P* = 0.001; Fig. 3A]. Beta dispersion of the bacterial communities was 1.9-fold greater for *Madracis* and 1.8-fold greater for other coral species combined compared to the CBL seawater (*P* adj < 0.0001 for both comparisons; Tukey HSD) but was similar between the bacterial communities of *Madracis* and other coral species (*P* adj = 0.91, Tukey HSD; Fig. S7). The mean Shannon diversity index of the CBL seawater bacterial community (2.85 ± 0.14) was ~1.6-fold and ~1.4-fold lower than that of *Madracis* (4.50 ± 0.25; *P* adj < 0.0001) and other corals (4.03 ± 0.08; *P* adj < 0.0001), respectively (Fig. 3B; Table S7). This pattern was similar for Simpson diversity, where the CBL seawater bacterial community (0.69 ± 0.03) was ~1.4-fold lower than that of *Madracis* (0.94 ± 0.01; *P* adj < 0.0001) and ~1.3-fold lower than that of other coral species (0.93 ± 0.01; *P* adj < 0.0001). The mean richness of bacterial genera was highest in the *Madracis* community (1,053.25 ± 17.55), compared to that of all other coral species combined (852.94 ± 36.94; *P* adj = 0.02, Tukey HSD), but was not significantly higher than the bacterial richness in the CBL seawater (878.71 ± 55.49; *P* adj = 0.06, Tukey HSD). The evenness of the bacterial communities was similar between *Madracis* (0.65 ± 0.04) and other coral species (0.60 ± 0.01; *P* = 0.40, Tukey HSD) but was significantly lower in the CBL communities (0.043 ± 0.03; *P* < 0.0001 for both comparisons).

A total of 1,099 bacterial genera across 61 classes were identified in the combined data set through our read-based analysis. Of these, 44 genera represented at least 1% of bacterial relative abundance within a given sample and are reported as the mean relative abundance of each genus in each coral species or the CBL seawater (Fig. 3C). *Madracis* bacterial communities were dominated by *Ruegeria* (8.38% ± 2.39%; mean ± SE) and *Sphingomonas* (6.14% ± 2.27%). In contrast, other coral species were, on average, dominated by the genera *Klebsiella* (8.60% ± 1.80%), Candidatus *Thioglobus* (7.33% ± 3.58%), and *Pseudomonas* (7.13% ± 0.57%). The 20 genera making the largest contribution to the dissimilarity between the bacterial communities of *Madracis* and other coral species accounted for 27% of the total variation between groups (Table S13), a level of explained variation consistent with complex microbial communities, where high richness and evenness often result in the variation being distributed across many taxa (78–80). Among these, 13 had a higher abundance in *Madracis*, including *Ruegeria*, *Hyphomicrobium*, and *Wenxinia*, with *Ruegeria* alone contributing nearly 5% of the dissimilarity. Seven genera had abundances at orders of magnitude lower levels in *Madracis*, including *Nitrosospira*, *Wenzhouxiangella*, and *Beijerinckia* (Table S13).

**Fig 4:**
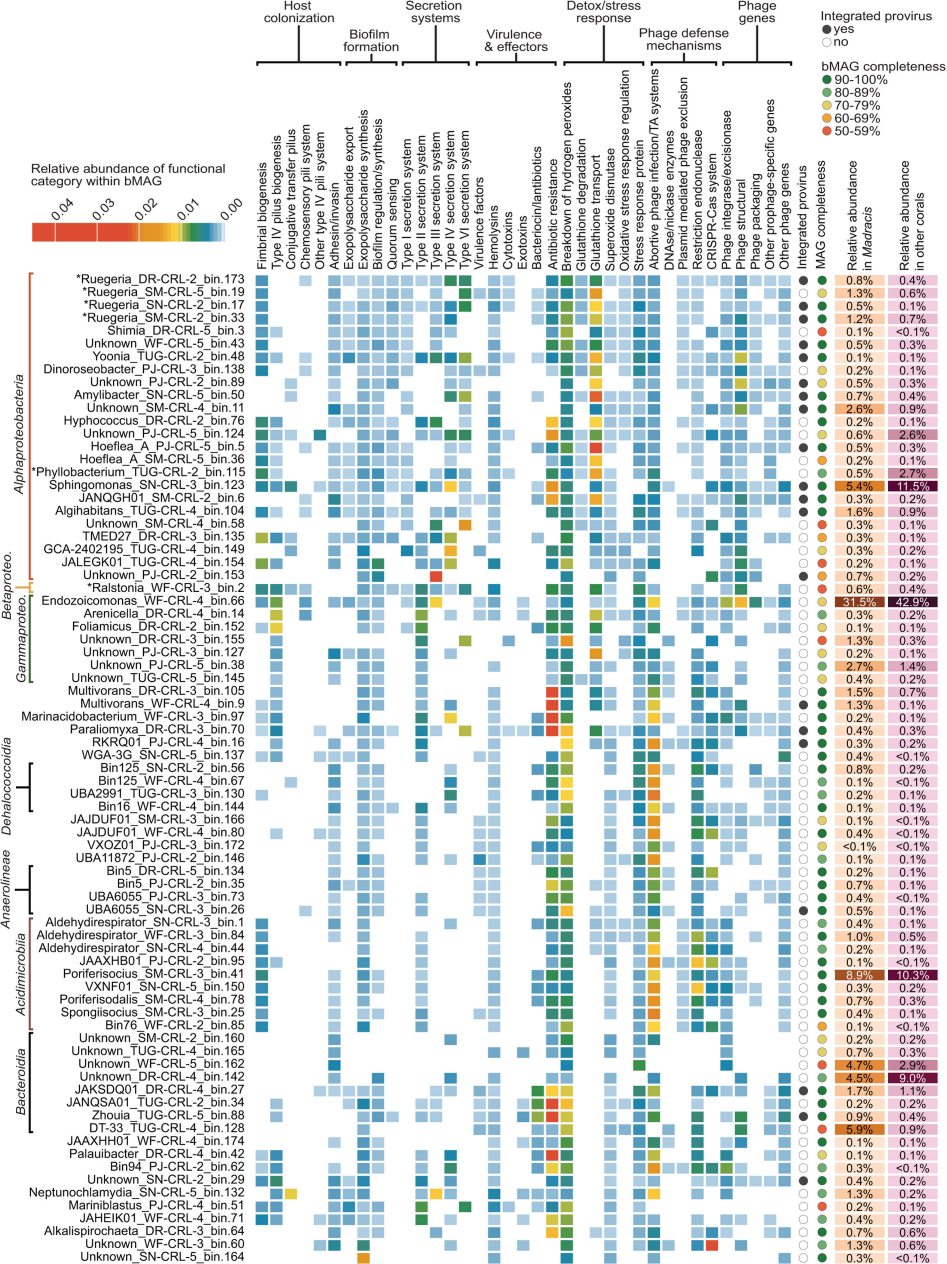
Symbiotic functions of bacterial MAGs from corals. Functional analysis of the 77 representative bMAGs. The color gradient from light blue to red represents the relative abundance of each functional category within a given bMAG, as determined by the sum of genes within each functional category. Genomes were annotated with the presence/absence of an integrated prophage, the completeness of the bMAG (ranging from 51.05% to 100%, with an average of 83.90%), and the average relative fractional abundance of each genome in *Madracis* and in other coral species. On the *y*-axis, genomes are sorted by their phylogenomic distance, as determined by GTBD-Tk. Classes with more than four representative genomes are described along the *y*-axis, and the full taxonomic classification of each bMAG can be found in Table S14.

A total of 2,112 refined bMAGs were dereplicated at a 95% similarity, yielding 77 representative species-level bins with 83.90% ± 1.63% completion and 1.91% ± 0.21% contamination (Fig. S8). These 77 bMAGs spanned 14 phyla, 19 classes, and 49 genera of bacteria (Fig. 4; Table S14). Seventy-eight percent (*N* = 60) of the bMAGs were identified to the genus level by GTDB-Tk, and 26% (*N* = 20) contained proviruses. All representative bMAGs were identified in the metagenomes of both *Madracis* and other coral species. The most abundant bMAG across all coral samples was *Endozoicomonas*_WF-CRL-4_bin.66, representing an average of 31.54% ± 7.02% of the bMAGs’ relative abundance in *Madracis* and 42.92% ± 5.35% in other corals. *Poriferisocius*_SM-CRL-3_bin.41 (8.87% ± 3.36% in *Madracis* and 10.31% ± 2.53% in other corals), *Sphingomonas*_SNAKE-CRL-3_bin.123 (5.42% ± 3.85% and 11.53% ± 2.91%), and a bin of the class *Bacteroidia* (Unknown_DR-CRL-4_bin.142; 4.49% ± 2.11% and 9.03% ± 2.60%) followed in abundance (Fig. 4). Although genome-size normalized fractional abundances were used to account for differences in genome length and sequencing depth, these values represent relative abundance among bacterial genomes that were successfully assembled into MAGs and dereplicated at the species level. As such, they likely represent a fraction of the true microbial diversity and are not reflective of absolute microbial abundance.

Functional analysis of the 77 representative genomes revealed 178,108 individual gene hits, spanning 8,845 unique gene products (Fig. 4). Gene products involved in fimbrial and pilus biogenesis, biofilm formation, secretion systems, effector/toxin production, and detoxification/stress responses were found throughout the bMAGs. Additionally, numerous genes related to phage defense mechanisms, particularly toxin-antitoxin (TA) systems and restriction endonucleases, were identified in the majority of the representative genomes. Confirmed prophages were detected in 26% of bMAGs (*N* = 20), but many more of these bacterial genomes encoded viral structural proteins (66%; *N* = 51) and phage integration genes (80%; *N* = 61), suggesting a potentially higher prevalence of prophages. Alternatively, these genes may represent laterally transferred viral genes or domesticated prophage remnants (34, 81). Three genera distinguishing the bacterial communities of *Madracis* and other corals in the SIMPER analysis had representative bMAGs, including *Ruegeria* (*N* = 4), *Ralstonia* (*N* = 1), and *Phyllobacterium* (*N* = 1). *Ruegeria* genomes were enriched in type IV and VI secretion systems, antibiotic resistance mechanisms, genes related to the breakdown of hydrogen peroxide, and glutathione transport genes (Fig. 4). Three of the four *Ruegeria* genomes were lysogens. The *Ralstonia* genome contained similar genes, along with a broader diversity of pilus biosynthesis genes related to eukaryotic host colonization. *Phyllobacterium* was relatively enriched in glutathione transport genes and had relatively fewer genes related to secretion systems. The genus *Sphingomonas,* the second most abundant genus in *Madracis*, had one representative bMAG encoding a prophage, type IV secretion system genes, antibiotic resistance genes, detoxification genes, and a variety of phage defense mechanisms. The presence of secretion systems and pilus in these individual bMAGs is consistent with the overall functional gene analysis of bMAGs: among the 15 most differentially abundant COG categories were those related to secretion systems and pilus biosynthesis (e.g., type II secretion/type IV pili, type III and V secretion systems), in addition to metabolism and energy production (e.g., TCA cycle, glyoxylate bypass; pyruvate oxidation; A/V-type ATP synthase), amino acid and cofactor biosynthesis (e.g., glutamine biosynthesis, lipoate biosynthesis, glycine cleavage), and nucleotide metabolism and modification (e.g., pyrimidine salvage, tRNA modification) (Fig. S9A). Secretion systems, phage defense mechanisms, and host attachment-related genes were more abundant in the bMAGs recovered from the other coral species.

All identified KEGG metabolism pathways were present in the bMAGs of both *Madracis* and other corals. Among the 15 most differentially abundant metabolism-related KEGG pathways, only two, sphingolipid metabolism (ko00600) and arginine and proline metabolism (ko00330), were more abundant in *Madracis* bMAGs than in bMAGs of other corals, though these differences were not statistically significant (Fig. S9B). The remaining pathways, including arachidonic acid metabolism (ko00590), nicotinate and nicotinamide metabolism (ko00760), pyrimidine metabolism (ko00240), retinol metabolism (ko00830), glycerolipid metabolism (ko00561), glutathione metabolism (ko00480), phenylalanine metabolism (ko00360), selenocompound metabolism (ko00450), riboflavin metabolism (ko00740), propanoate metabolism (ko00640), cysteine and methionine metabolism (ko00270), inositol phosphate metabolism (ko00562), and cofactor metabolism, constituted a greater mean abundance in other corals, but only nicotinate and nicotinamide metabolism (ko00760) was statistically significant.

In addition to analyzing the bMAG communities, we also examined the broader bacterial contig data set, which included both binned and unbinned bacterial contigs. Among the 15 most differentially abundant COG categories identified in the bacterial contigs, only the NADH dehydrogenase and Aminoacyl-tRNA synthetases categories showed a statistically significant difference (Fig. S10A). Similarly, only the glycerophospholipid metabolism pathway (ko00564) showed a statistically significant difference between *Madracis* and other coral-associated bacterial contig communities (Fig. S10B). Despite the lack of significant differences at the pathway level, the overall metabolic diversity (KEGG pathways; Fig. S11) and gene product diversity of bacterial contigs (as measured by Shannon and Simpson indices, and gene richness), as well as the overall functional redundancy, were consistently higher in *Madracis*-associated bacterial contigs compared to those of other coral species (Fig. S12).

### Viral communities associated with *Madracis*

**Fig 5:**
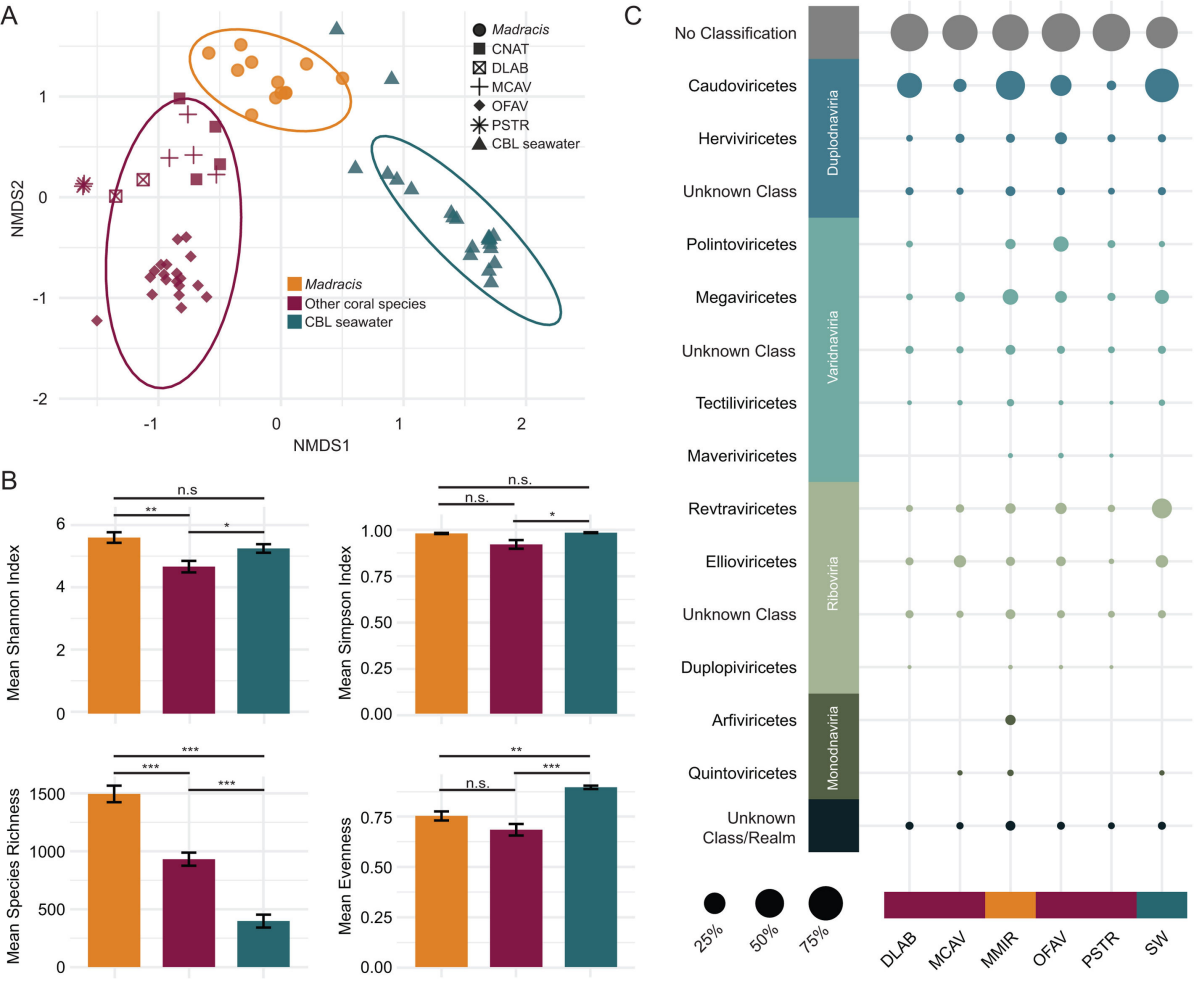
Viral community dissimilarity between *Madracis*, other coral species, and coral boundary layer (CBL) seawater. (**A**) Non-metric multidimensional scaling (NMDS) plot based on Bray-Curtis distances calculated from the relative abundance of viruses with 999 permutations. Ellipses denote a 95% confidence interval for the sample types. (**B**) Mean diversity indices of viral communities, including Shannon Index, Simpson Index, Evenness, and Richness. Significance codes: 0, ***; 0.001, **; 0.01, *. (**C**) Relative fractional abundances of viral classes in corals and coral boundary layer (CBL). The size of the circles indicates the relative abundance of each class, and the color of the bubbles indicates viral realms. Coral species names are abbreviated as follows: *Colpophyllia natans*, CNAT; *Diploria labyrinthiformis*, DLAB; *Montastraea cavernosa*, MCAV; *Madracis mirabilis*, MMIR; *Orbicella faveolata*, OFAV; *Meandrina meandrites*, MMEA; and *Pseudodiploria strigosa*, PSTR.

Viral communities were significantly different between *Madracis*, other coral species, and the CBL [F(2, 61) = 10.126, *P* = 0.001; Fig. 5A]. Beta dispersion between groups only differed between other coral species and the CBL (*P* = 0.01, Tukey HSD) (Fig. S7). The Shannon diversity of viral communities in *Madracis* (5.53 ± 0.17) and the CBL (5.18 ± 0.13) were higher than that of other coral species (4.62 ± 0.18; Fig. 5B; Table S7), while the Simpson diversity of viral communities was similar between *Madracis* (0.99 ± 0.003) and the CBL (0.99 ± 0.002) or other coral species (0.93 ± 0.02). Viral community richness differed among groups [F(2, 61) = 58.85, *P* = 5.79e−15] and was 1.6- to 3.8-fold higher in *Madracis* (1,500.25 ± 71.91) than in other corals (934.45 ± 56.42) and the CBL (397.95 ± 56.03). The evenness of the viral communities was similar between the two coral groups (0.76 ± 0.02 for *Madracis* and 0.69 ± 0.03 for other coral species) and lower than the CBL [0.90 ± 0.01; F(2, 61) = 19.98, *P* = 2.12e−07].

A total of 2,820 dereplicated and quality-filtered viral genomes and genome fragments spanning four viral realms and 11 viral classes were identified in coral and CBL communities (Fig. 5C). The viral class *Caudoviricetes* dominated in *Madracis*, comprising 24.56% ± 5.06% (mean ± SE) of the community, followed by *Megaviricetes* (3.33% ± 0.80%) and *Revtraviricetes* (1.59% ± 0.27%). In other coral species, *Caudoviricetes* viruses were also the most abundant class, though at a 2.2-fold lower relative abundance (11.26% ± 1.59%). These were more closely followed by viruses from the classes *Polintoviricetes* (5.30% ± 0.55%), *Ellioviricetes* (4.36% ± 1.46%), and *Herviviricetes* (2.53% ± 0.26%). Of these viruses, 1,193 significantly contributed to the between-group differences, 179 of which explained 75% of the total dissimilarity. The 20 viruses contributing most to between-group differences, accounting for 22% of total dissimilarity, included viruses from the classes *Caudoviricetes*, *Megaviricetes*, and *Polintoviricetes*, and nine viruses that could not be taxonomically classified (Table S15). Of these, eight were predicted to infect *Endozoicomonas*.

**Fig 6:**
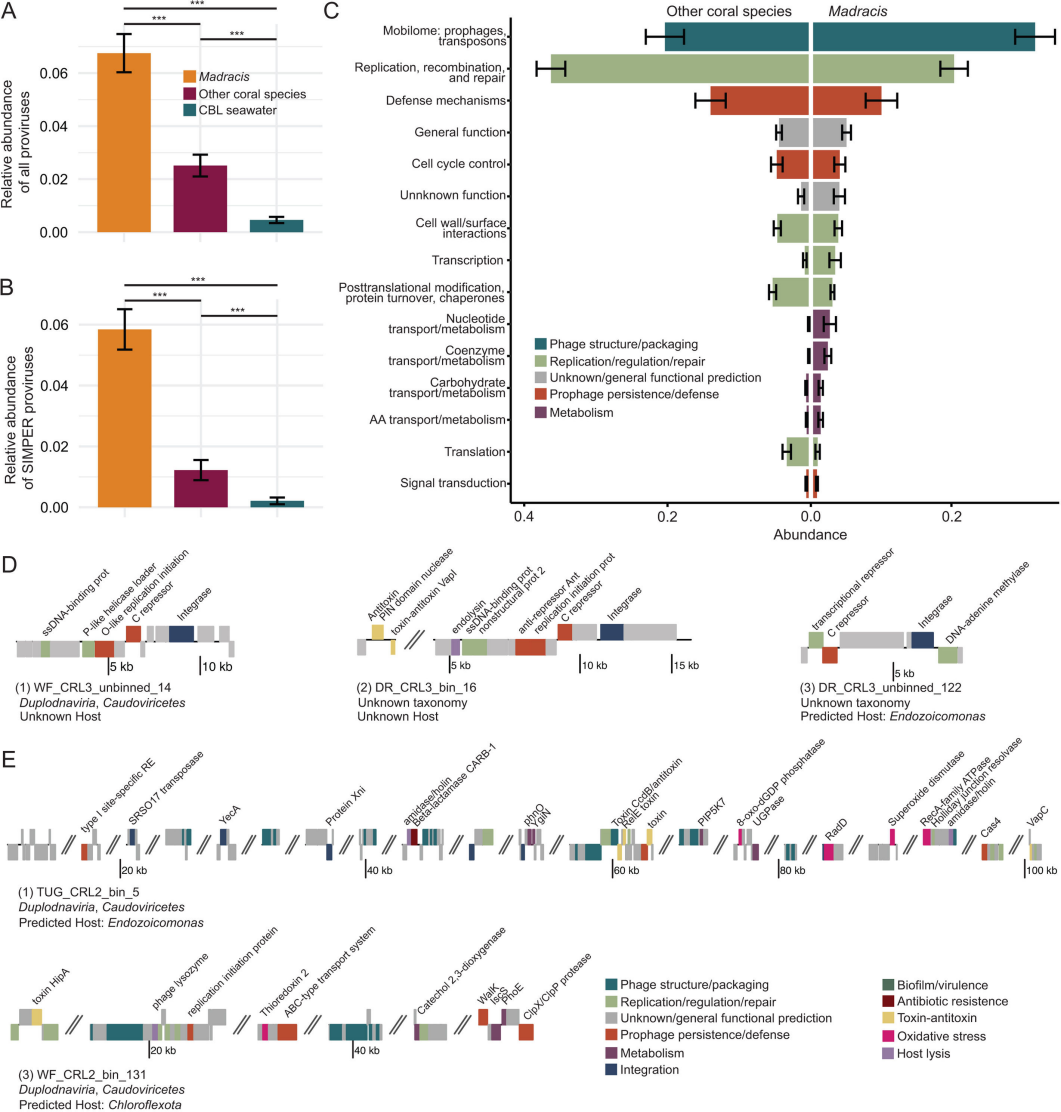
Genomic content of indicator viruses and proviruses of the *Madracis* holobiont. (**A**) Relative abundance of all proviruses and (**B**) relative abundance of proviruses identified by SIMPER as significantly distinguishing the *Madracis* viral community from that of other corals*.* Significance code: 0, ***. (**C**) The 15 most abundant COG categories encoded by proviruses in other coral species (left) and *Madracis* (right). (**D**) Genome maps of *Madracis* indicator viruses encoding integrases. (**E**) Genome maps of two high-quality *Madracis* proviruses carrying metabolic or symbiosis-related genes.

Proviruses accounted for an average of 6.75% ± 0.72% of all viruses in *Madracis*, significantly more than the 2.51% ± 0.41% of viruses in other coral species and 0.46% ± 0.11% of viruses in the CBL (Fig. 6A; Table S8). Among the 1,193 viruses that significantly contributed to the between-group differences, proviruses accounted for an average of 5.84% ± 0.66% of viruses in *Madracis*, which was significantly greater than in other coral species (2.62% ± 0.69%) and the CBL (0.21% ± 0.11%) (Fig. 6B; Table S8). Proviruses from the two coral groups encoded genes classified as COG categories “Mobilome: prophages and transposons,” “Replication, recombination, and repair,” and “Defense mechanisms” (Fig. 6C). Lower abundance metabolism-related functional categories, including “Nucleotide transport and metabolism,” “Coenzyme transport and metabolism,” and “Amino acid transport and metabolism” were enriched in *Madracis* proviruses relative to those of other corals. The categories “Cell wall/surface interactions,” “Posttranslational modification, protein turnover, chaperones,” and “translation” were enriched in the proviruses of other corals.

Forty-four viruses were significantly associated with *Madracis* at ≥95% specificity and fidelity (Table S16). High specificity values (e.g., A ≥ 0.95) represent viruses that are primarily or exclusively found in *Madracis* samples, and high fidelity values (e.g., B ≥ 0.95) represent viruses that are found across most or all *Madracis* samples. Of the indicator viruses that had successful host predictions (*N* = 5), *Endozoicomonas* (*N* = 4) and *Vibrio* (*N* = 1) hosts were identified. Among the 44 indicator viruses, 10 with 100% specificity and fidelity in *Madracis* were selected for further genome analysis. Three encoded integrases, indicating that they may have the ability to integrate into their host genome, although they were not identified as integrated in a bMAG (Fig. 6D). These temperate indicator viruses of *Madracis* primarily encode genes for genome replication and capsid structure. DR_CRL3_bin_16, the largest virus of the three, was an exception, encoding genes related to toxin-antitoxin systems. While only one of these viruses could be taxonomically classified (WF_CRL3_unbinned_14; *Caudoviricetes*), all three viruses also carry a phage repressor protein C, which contains Cro/C1-type HTH and peptidase S24 domains. This regulator of the lysogenic-lytic switch is characteristic of lambda phage, a temperate member of *Caudoviricetes* (82–84). Only DR_CRL3_unbinned_122 had successful host prediction, with *Endozoicomonas* identified as the putative host. In addition to these viruses, two high-quality prophage genomes encoded auxiliary gene products for antibiotic resistance (*CARB-1* beta-lactamase), metabolism (*phnO*, *ygiN,* catechol 2,3-dioxygenase, *iscS*, *phoE*), prophage and host persistence (toxin-antitoxin systems, *recA*), and resilience under oxidative stress (8-oxo-dGDP phosphatase, *radD*, superoxide dismutase, thioredoxin 2) (Fig. 6E). Based on the annotation of their bacterial flanking regions, these prophages were predicted to infect *Endozoicomonas* (82.2% identity over 34% query coverage, E-value = 1e−68) and *Chloroflexota* (90.72% identity over 83% query coverage, E-value = 0.0).

## DISCUSSION

### Coexistence with high microbial densities

In Curaçao, *Madracis mirabilis* forms expansive colonies composed of many tightly spaced branches, expanding on degraded and increasingly microbialized reefs where other corals have not achieved similar success (Fig. 1). Microscopy results revealed significantly denser viral and microbial communities and increased VMRs within *Madracis* branches (IB) compared to the boundary layer (BL) samples (Fig. 2). The natural branching complexity of *Madracis* forms a stagnant region in the center of the colony at high water flow (85), likely contributing to the increased densities of bacteria and viruses observed within the branches. Elevated microbial densities on reefs can drive coral mortality via oxygen depletion and the formation of hypoxic zones (86), as well as increased presence of microbial pathogens (10). The natural structure of *Madracis*, which harbors areas of dense microbial biomass, may contribute to *Madracis*’s natural resistance to reef microbialization that is commonly associated with pollution and reef degradation, as observed in the Curaçao reefs where *Madracis* occurs (10). Mechanisms such as enhanced tolerance to oxygen fluctuations and hypoxia, pathogen defense mechanisms, or coral host physiological traits may enable resistance to high bacterial densities, contributing to the persistence and success of *Madracis* in Curaçao. Whether these highly dense microbial and viral communities actively confer resilience or are a byproduct of other host traits or environmental factors remains uncertain.

### Disease resistance mechanisms and community structure

*Madracis* bacterial communities were distinct from those of other corals, showing dominance by *Ruegeria* and *Sphingomonas*, whereas other coral species were enriched in *Klebsiella*, Candidatus *Thioglobus*, and *Pseudomonas* (Fig. 3C). SIMPER analysis further identified *Ruegeria* and *Wenxinia* (family *Rhodobacteraceae*) as key genera differentiating *Madracis* from other coral species, with a relative depletion of *Nitrosospira* and *Beijerinckia* (Table S13). We identified four distinct *Ruegeria* bMAGs, three of which were enriched in type VI secretion system (T6SS) genes and effectors that may contribute to *Madracis*’s success (Fig. 4). These genes are involved in interbacterial competition that may suppress invading pathogens, contributing to disease resistance in *Madracis*. Hcp proteins found in these bMAGs can function as effector toxins, targeting competing bacteria and promoting microbial dominance of beneficial taxa (87). Representatives of *Ruegeria* have been described in association with healthy corals compared to bleached colonies (88), and certain *Ruegeria* strains have been shown to inhibit coral pathogens like *Vibrio coralliilyticus* through unknown mechanisms (89, 90). Future studies should test if *Ruegeria*’s Hcp proteins can suppress *Vibrio coralliilyticus* and promote coral health in controlled conditions.

Bacterial community compositions differed between read-based (Fig. 3) and bMAG (Fig. 4) analyses, reflecting known biases of genome-resolved metagenomics, which tends to recover the high-abundance strains that are easier to assemble (51, 91, 92). This leaves out low-abundance, strain-diverse, or short-genome taxa, which are not recovered as high-quality bMAGs. Additionally, our bMAGs likely reflect strains, while the read-based analysis was performed at the genus level. As a result, a genus with many micro-diverse strains may not be accurately reflected in the bMAG data. Conversely, some abundant bMAGs belonged to genera displaying low read-based abundance at genus level classifications, likely due to the lack of adequate representation of these taxa in the reference databases used in the read analysis (51). For example, a single *Endozoicomonas* genome was the most abundant bMAG, but it was not detected in the genus-level read-based profiles. This illustrates how read-based methods that rely on reference databases can overlook ecologically important but novel taxa and highlight the strength and complementarity of the MAG approach. On the other hand, *Ruegeria* was abundant in both the read-based and bMAG approach, indicating that it was both abundant in our samples and well-represented in reference databases, despite the high species/strain level diversity within our *Ruegeria* bMAGs. Furthermore, recent work using 16S rRNA gene sequencing to characterize the microbiome of *Madracis* in urban versus protected reef habitats revealed a temporally stable community dominated by *Endozoicomonas* and *Vibrionaceae* (93). These genera were not prominent in our read-based data, but *Endozoicomonas* was represented in our bMAG analysis, highlighting the importance of integrating different approaches to generate a more complete picture of microbial community structure.

Among the viral communities, *Madracis* also exhibited higher diversity and richness than all other corals. *Caudoviricetes* (tailed phages) were the most abundant viral class across all corals, consistent with previous studies of coral viral communities, and were significantly more abundant in *Madracis* than in other corals (45, 94) (Fig. 5C). Tailed phages play roles in regulating bacterial populations, nutrient cycling, and genetic exchange (39). The elevated presence of these bacteria-infecting viruses in *Madracis* indicates that this species’ viral community may be more prominent in modulating bacterial community dynamics than in other corals. These bacteriophages could help control pathogen invasion via lytic infections, aligning with the Bacteriophage Adherence to Mucus model (38). Resolving individual virus-host linkages remains a key future direction to uncovering the putative roles of these viruses in pathogen defense. We reported host predictions for SIMPER and indicator viruses, as well as those selected for genome annotation (Fig. 6D and E; Table S15); however, only a few viral genomes yielded successful host assignments. Among these, the majority were matched to *Endozoicomonas*, an important coral symbiont recognized for correlation with coral health (95–97). With potential roles in holobiont nutrient cycling (97, 98), the functional capacity of *Endozoicomonas* may be influenced by frequent viral infection. Integration of additional data sets, such as Hi-C proximity ligation or complementary host prediction tools, could expand host linkage across the data set.

*Madracis* microbiomes harbored a significantly higher abundance of prophages compared to other coral species (Fig. 6A and B). Genes related to TA systems were prevalent in prophage genomes, highlighting a potential mechanism for *Madracis* microbiomes to resist bacteriophage superinfection and enhance bacterial stability under stressful conditions (99, 100). This aligns with previous work suggesting that TA systems enable bacterial “persisters” to survive harsh conditions, such as antibiotic exposure or nutrient depletion, by temporarily shutting down key processes (e.g., bacteriophage propagation) (101). Prophages infecting beneficial bacteria, such as *Ruegeria*, could help stabilize the bacterial community by preventing competitive displacement and increasing host resilience. Additionally, prophages encoding genes for antibiotic-resistance proteins, such as *CARB-1 beta-lactamase*, may confer protection against antibiotics and antimicrobial compounds produced by competitors, further enhancing bacterial persistence within the coral holobiont (102). Notably, three of the four *Ruegeria* bMAGs contained integrated proviruses, further supporting their role in microbiome resilience.

### Bacterial adaptations to nitrogen loading, organic matter inputs, and pollutants

Consistent with prior observations, coral-associated bacterial communities exhibited greater Shannon and Simpson diversity compared to seawater collected around the corals, underscoring the distinctions between these free-living and host-associated communities (103, 104) (Fig. 3B). Among corals, *Madracis* harbored a significantly richer bacterial community at the genus level than all other coral species combined. Microbiome diversity has been implicated in host responses to changing environments (105, 106). In the context of the success of *Madracis* in Curaçao, these highly diverse communities could provide advantages, such as functional redundancy, metabolic versatility, and resilience, in the face of environmental stress (107–109). In fact, *Madracis*-associated bacterial communities exhibited a greater diversity of gene products and higher functional redundancy than those of other coral species, suggesting a more versatile genomic repertoire, as well as increased functional resilience to community disturbances (Fig. S12).

These patterns also raise questions about the role of the coral host in shaping microbial diversity. Coral-associated microbiomes may be influenced by a combination of environmental exposure, vertical or horizontal microbial transmission, and host selection of microbiome members. Traits such as mucus composition, immune recognition, or symbiont filtering mechanisms may enable some hosts, like *Madracis*, to support more diverse or stable microbial communities (110, 111). Future work combining host transcriptomics, microbial functional profiling, and manipulative experiments (e.g., reciprocal transplants) will be important to disentangle the relative contributions of host identity and environment in structuring the microbiome.

The *Ruegeria* genomes encoded genes related to nutrient cycling, organic matter degradation, and interbacterial competition. Consistent with other *Ruegeria* (112, 113), these genomes also contained genes associated with nitrogen fixation and metabolism (e.g., *fixH*, *ntrB*, *glnB*/*glnK*), as well as organic matter and pollutant degradation (e.g., *ssuD*, cytochrome P450, catechol 2,3-dioxygenase). The presence of these genes suggests that *Ruegeria* may play a role in the bioavailability of nutrients for the coral host and its algal symbionts while also impacting organic matter availability for copiotrophic bacteria.

Another genus found to be highly abundant in *Madracis* was *Sphingomonas* (class *Alphaproteobacteria*), which has recognized roles in the degradation of complex organic compounds, production of UV-protective carotenoids, and bioremediation (114–116) (Fig. 4). In degraded reef environments, microbial communities must adapt to increased organic pollution, oxidative stress, and shifting nutrient dynamics. A single representative *Sphingomonas* bMAG encoded type IV secretion systems (T4SS), antibiotic resistance genes (e.g., *ampG*, β-lactamases), and multiple genes linked to bioremediation (e.g., catechol 2,3-dioxygenase, ring-hydroxylating dioxygenases, and arsenical resistance genes). This suggests that *Sphingomonas* contributes to detoxification processes and holobiont resilience in degraded reef environments, although its presence is not unique to *Madracis*. T4SS can facilitate horizontal gene transfer, potentially spreading antibiotic resistance and other contact-based interactions, indicating broader ecological functions (117).

### Prophage contributions to bacterial metabolic diversity

As agents of gene transfer, proviruses contribute to the genomic flexibility, adaptability, and resilience of bacterial communities (39). Rather than causing lysis of bacterial populations, temperate viruses can integrate as dormant prophages, which may help maintain a stable and beneficial microbiome by introducing functional genes that enhance bacterial survival under environmental stress (118). The enrichment of prophages in *Madracis* suggests that viral-mediated gene transfer may influence bacterial functions with direct implications for the coral host, particularly in degraded reef environments. Relative to other corals, *Madracis*-associated prophages encoded a greater diversity and proportion of genes related to metabolic functions (Fig. 6C), suggesting they may play roles in diversifying the metabolic potential of *Madracis*-associated bacteria. The prophage enrichment aligns with the enrichment of members of the *Alphaproteobacteria* in this coral, such as *Sphingomonas*, since these *Alphaproteobacteria* have been shown to carry a large number of diverse prophages (43, 119, 120). Based on the prophage modification of host physiology in other systems (121), we speculate that *Madracis*-associated prophage genes may modulate bacterial metabolism in the coral. Of the annotated proviruses (Fig. S6E), one was predicted to infect *Endzoicomonas* and the other, *Chloroflexota. Endozoicomonas* is widely considered an abundant and beneficial symbiont of corals, but the role of *Chloroflexota* is less clear (97, 98, 122). While *Chloroflexota* is typically much less abundant, members of this phylum, particularly *Anaerolineae*, are consistently detected in coral microbiomes, often in low-oxygen environments (122, 123). The roles of both taxa in nutrient cycling may be impacted by viral integration. Their prophages encoded genes, including *phnO*, which is involved in the metabolism of alternative phosphorus sources and could be beneficial in situations where nitrogen loading results in phosphorus limitation (124, 125). Additionally, genes encoding for enzymes like *ygiN*, which help detoxify quinone-like compounds, and catechol 2,3-dioxygenase, which facilitates the breakdown of aromatic compounds, can further contribute to toxin degradation (126, 127). Prophages encoding cysteine desulfurases may also be involved in sulfur metabolism and oxidative stress responses (128). Genesrelated to ROS detoxification (e.g., 8-oxo-dGDP phosphatase*, RadD,* superoxide dismutase, and thioredoxin 2 likely help maintain bacterial function under oxidative stress (129–132). Alternatively, these genes may play a role in protecting intracellular bacteria from oxidative stress caused by Symbiodiniaceae photosynthesis (133).

### Conclusions and future directions

Our study highlights the unique microbial traits of the *Madracis mirabilis* holobiont that may contribute to its success in degraded reef environments where other coral species struggle. The dense microbial and viral communities within *Madracis* colonies may confer pre-adaptation to microbialization and, therefore, provide resilience against reef-level degradation that is accompanied by high bacterial cell densities. The dominance of specific microbial genera, such as *Ruegeria*, and the high functional diversity and redundancy, with functional genes related to pathogen suppression, nutrient cycling, and pollutant degradation, indicate that *Madracis* may rely on its microbiome to support its survival in eutrophic conditions. Additionally, the high abundance of prophages and high viral diversity in *Madracis* suggest that viral-mediated gene transfer plays a role in microbiome genomic flexibility via lateral gene transfer. These findings underscore the potential of *Madracis* to serve as a model for understanding coral resilience in the face of anthropogenic reef degradation. Identifying microbial taxa and viral traits in a resilient coral species like *Madracis* may serve as a foundation for forecasting coral community trajectories. Features such as high functional redundancy, enrichment of certain beneficial taxa, or genomic profiles with the capacity for stress mitigation could be tested as indicators of resilience potential. Future studies could evaluate these biomarkers across reef systems and integrate them into predictive frameworks that link shifts in community or functional composition to coral stress responses or survival outcomes under different environmental contexts. Furthermore, identifying microbial traits enriched in successful corals in degraded habitats offers a starting point for exploring microbiome-based interventions, such as microbiome engineering strategies, where they may serve as targets for probiotic development or for prioritizing microbial assemblages.

## Supplementary Material

Reviewer comments

## Data Availability

Coral and CBL seawater metagenomic sequence data for this project are available on NCBI (PRJNA1214393 and PRJNA1061506). The code used to conduct this analysis is publicly available on GitHub (https://github.com/Silveira-Lab/Wallace_Madracis_Holobiont). R code and data are available on figshare (doi: 10.6084/m9.figshare.28216004), along with assembled viral genomes (doi: 10.6084/m9.figshare.28259492), bacterial MAGs (doi: 10.6084/m9.figshare.30148714), QC reports (doi: 10.6084/m9.figshare.28215692), and mapping data (doi: 10.6084/m9.figshare.29821319).

## SUPPLEMENTAL MATERIAL

Supplemental Material msystems.01208-25-s0001.docx Supplemental methods and results; Figures S1 to S12; captions for supplemental tables.

Supplemental Tables msystems.01208-25-s0002.xlsx Tables S1 to S16.

OPEN PEER REVIEW reviewer-comments.pdf An accounting of the reviewer comments and feedback.

ASM does not own the copyrights to Supplemental Material that may be linked to, or accessed through, an article. The authors have granted ASM a non-exclusive, world-wide license to publish the Supplemental Material files. Please contact the corresponding author directly for reuse.
