# Supplementary material for: High microbial diversity, functional redundancy, and prophage enrichment support the success of the yellow pencil coral, Madracis mirabilis, in Curaçao’s coral reefs
Source: mSystems. 2025 Oct 16;10(11):e01208-25. doi: 10.1128/msystems.01208-25 (PMC12625765; doi:10.1128/msystems.01208-25)
Supplement: Supplemental Material — Supplemental methods and results; Figures S1 to S12; captions for supplemental tables. [file msystems.01208-25-s0001.docx]

**Supplementary Text**

**Supplementary Methods:**

***Imaging and analysis of coral interaction outcomes***

For imaging of coral interactions, a transect tape (for scale reference) was placed in the direction of the current at an average of 7.54 ± 2.03 m depth (mean, SD), and the patch and interaction zone were photographed for quantification of the interaction zone outcomes (N = 11). At one of the sites, the photographic documentation was insufficient to enable accurate quantification of interaction outcomes, and data from this site were excluded.

To quantify and qualitatively characterize the interactions between *Madracis* and other coral species, images from each site were processed using ImageJ (Schneider, 2012). A transect tape placed in the direction of the current was used as a scale reference to measure the entire perimeter of the *Madracis* patch and interacting coral colony and categorize sections of the perimeter based on the neighboring substrates/organisms with which the coral colony was interacting. Perimeter interactions were first categorized into one of two groups, “Interaction zone (IZ)*”* or “all other interacting substrates (SUB)” (including algae and algal turfs, rubble, and/or other interacting coral species) (Fig. S3A). Within each of those groups, the interaction perimeter was also characterized by outcome (winning, losing, or neutral) using the interacting coral as the reference following the method described in George et al., 2021 (Fig. S3B). Here, outcomes were classified as losing, where *Madracis* or other substrates are overgrowing or causing visible damage to the interacting coral; neutral, where neither coral nor other substrates are overgrowing or damaging one another; or winning, where the interacting coral is overgrowing or causing visible damage to the neighboring *Madracis* or other substrates (George et al., 2021). Where *Madracis* was overtaking the interacting coral along over 60% of the interaction margin (N = 6), these corals were characterized as overall losing to *Madracis*. Where the interacting coral was overtaking *Madracis* along over 60% of the interaction margin (N = 3), these corals were characterized as winning against *Madracis*.

**Supplementary Results/Discussion:**

***Interaction outcomes***

The mean percentage of other coral species’ losing perimeter in interactions with *Madracis* was 56.83% (SE = 14.04%; N = 11), ranging from 0% to 100% of the interacting perimeter for individual coral colonies (Fig. S3C). The mean percentage of winning perimeter was 29.13% (SE = 13.84%), and the mean percentage of neutral perimeter was 14.03% (SE = 7.60%) across all interactions. Among interactions with other benthic substrates than *Madracis*, corals had more neutral (37.45% ± 12.86%; Mean, SE), less losing (39.33% ± 11.81%), and less winning (22.24% ± 11.76%) perimeter than they had against *Madracis* (Fig. S3D). In classifying the overall interaction outcomes based on a 60% cut-off, only a single coral interacting with *Madracis* (PSTR_DB) was characterized as neutral, three as winning, and seven as losing. *Montastraea cavernosa* (MCAV), a physically aggressive species, was the only interacting coral species to outcompete live *Madracis* along every interaction (N = 2). While one *Colpophyllia natans* (CNAT) was winning along 100% of its shared perimeter with *Madracis*, two other corals of the same species were characterized as overall losing interactions. *Pseudodiploria strigosa* (PSTR; N = 2) was another species that exhibited a variable interaction outcome with *Madracis* across samples. Overall, the only consistent species-specific interaction outcome was with *M. cavernosa*.

***Indicators of interaction outcomes***

We investigated potential relationships between the bacterial and viral community compositional differences and winning vs. losing interactions between *Madracis* and other coral species. When grouped by winning or losing interaction outcome, there was no separation between groups for bacterial (F(1, 22) = 0.896, p = 0.511) or viral (F(1, 22) = 1.571, p = 0.139) communities (Fig. S4). Ratios of *Bacteroidetes* to *Bacillota* (previously known as *Firmicutes*) were also calculated, as these ratios are a commonly used marker of dysbiosis in other systems (71-73) and have been shown to predict interaction outcomes between coral and turf algae (Roach et al., 2020). Here, the interaction zone samples did not have a significantly different relative abundance of *Bacteroidetes* or *Bacillota* than in coral samples, and the mean *Bacteroidetes* to *Bacillota* ratios did not significantly differ between winning (4.99 ± 1.62; Mean, SE) and losing (4.17 ± 2.03; Mean, SE) corals (Fig. S5).

***Viral and bacterial communities could not predict interaction outcome***

NMDS ordination of bacterial and viral communities showed no statistically significant differences between communities of winning vs. losing corals. These results may indicate that species-specific differences are more important in determining interaction outcomes. Alternatively, the resolution of sampling in this study and the determination of interaction outcomes (based on a 60% threshold of the entire interaction with *Madracis*) may not have been fine-scale enough to capture differences. Previous work on coral-algae interactions identified an overrepresentation of *Bacteroidetes* and an underrepresentation of *Firmicutes* (now *Bacillota*), at the interface where corals and algae interact (Roach et al., 2020). These ratios also predicted whether coral or algae won in direct competition, with *Bacteroidetes* enriched in losing corals and *Firmicutes* (*Bacillota*) depleted in losing coral samples (Roach et al., 2020). We did not observe the same trends in our dataset, consistent with the hypothesis of a distinct microbial community assembly and dynamics in *Madracis* compared to most other coral species in the studied reefs.

**Supplementary Figures:**

**
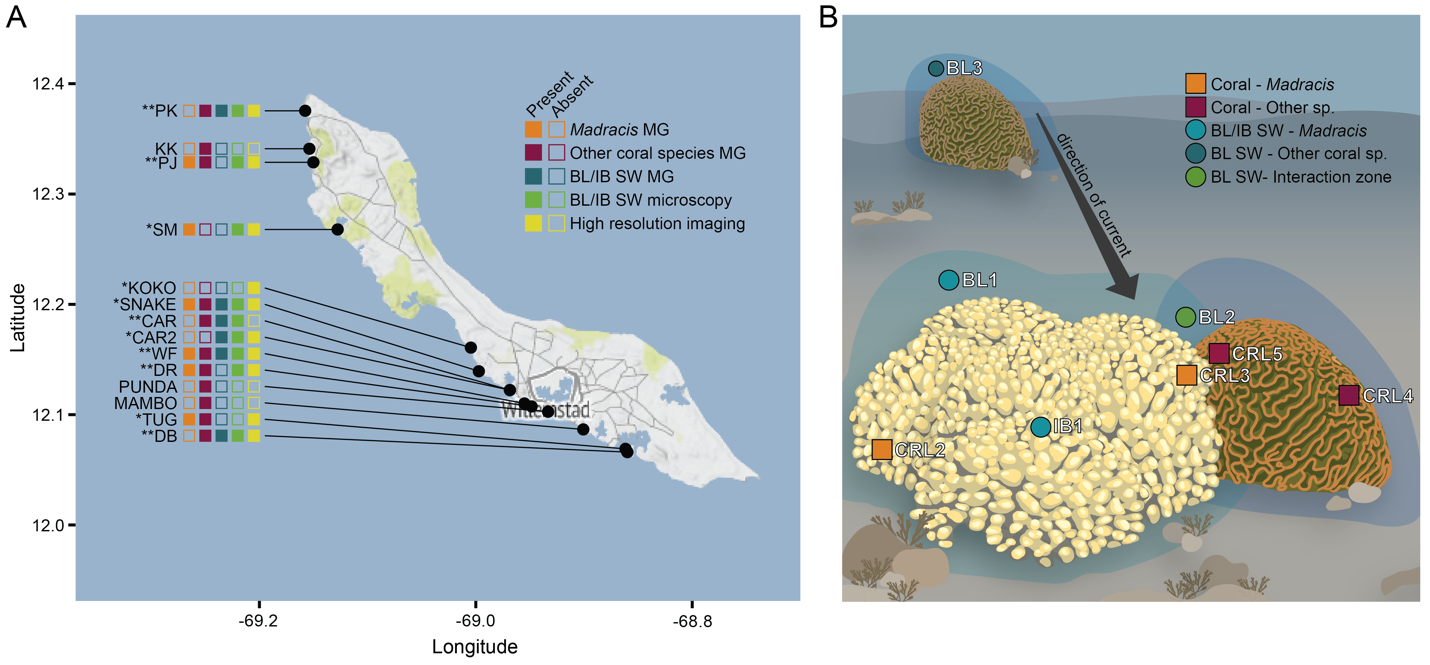
**

**Figure S1 | Sampling locations and layout.** (A) Map of Curaçao indicating the sampling locations (black circles) and the sample types at each site: metagenomic samples, microscopy samples, and high-resolution images of coral-coral interactions. Filled boxes indicate that the sample type was collected and successfully sequenced or analyzed; empty boxes are indicative of the sample types that were either not collected or not selected for metagenomic sequencing along each of the 14 sites. Asterisks indicate the sampling year, where “*” indicates that the site was visited only in 2022, and “**” indicates that the site was visited in both 2021 and 2022. (B) Conceptual sampling schematic for 2022 sampling of *Madracis* interactions. Coral samples (represented by squares) were collected at the edge of the *Madracis* patch (CRL2), at the interaction zone (CRL3 and CRL5), and at the far edge of the interacting coral (CRL4). Seawater samples were collected from within the *Madracis* fingers (IB1), the *Madracis* boundary layer (BL1), the interaction zone boundary layer (BL2), and the boundary layer of an upstream coral of the same species as the interacting coral (BL3).

**
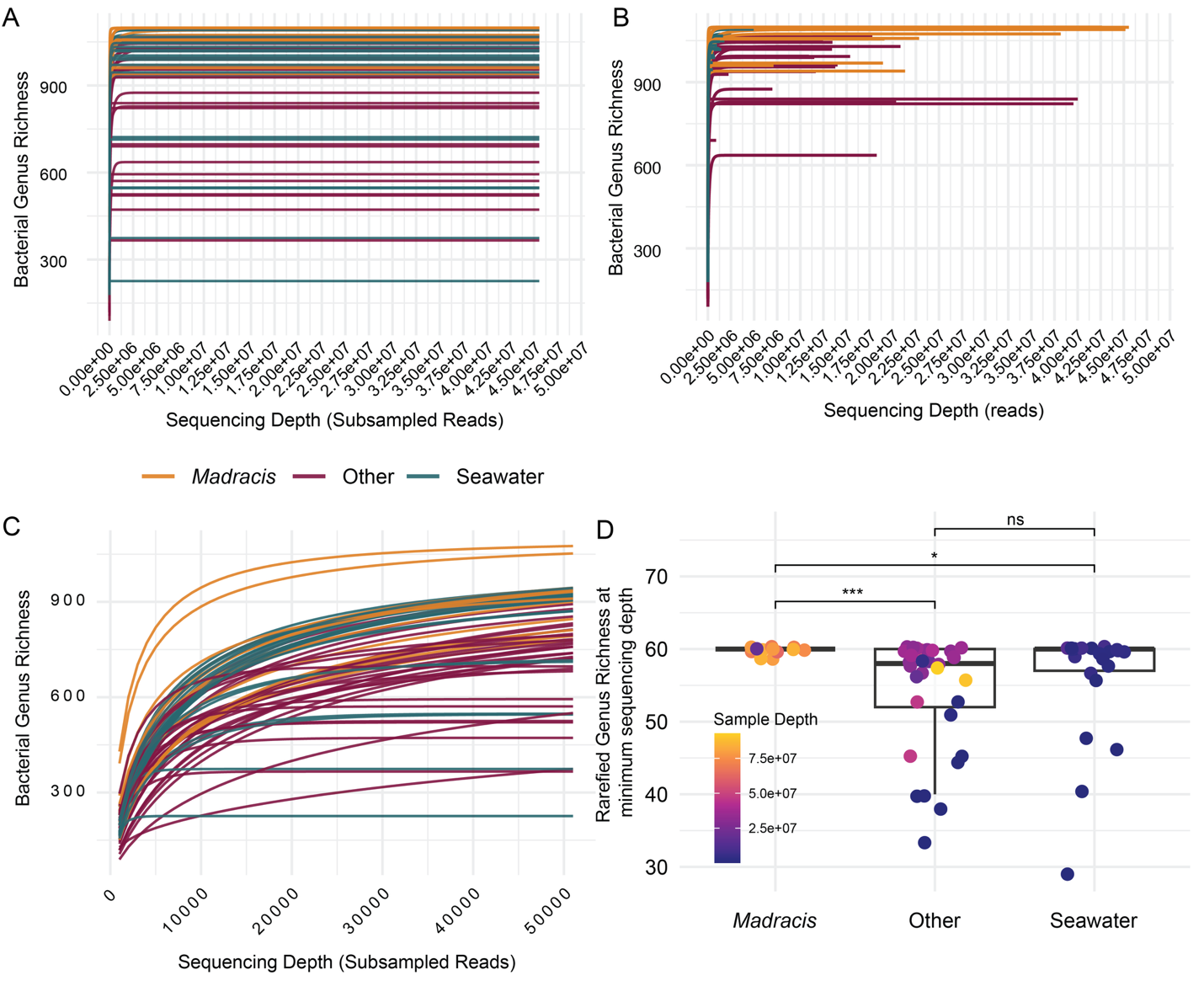
**

**Figure S2 | Collectors’ curves of bacterial genera recovery by sequencing depth.** (A) Collectors’ curves showing observed richness of bacterial genera across all samples, with curves extended to the maximum sequencing depth for comparison. (B) Identical plot to (A), but each sample's curve is truncated at its actual sequencing depth. (C) Collector’s curves rarefied to the minimum sample depth (51,238 reads) to better visualize diversity trends across samples. (D) Boxplot of bacterial genera richness after rarefaction, where individual data points are color-coded by sample sequencing depth (Significance codes: 0 ‘***’, 0.001 ‘**’, 0.01 ‘*’).

**
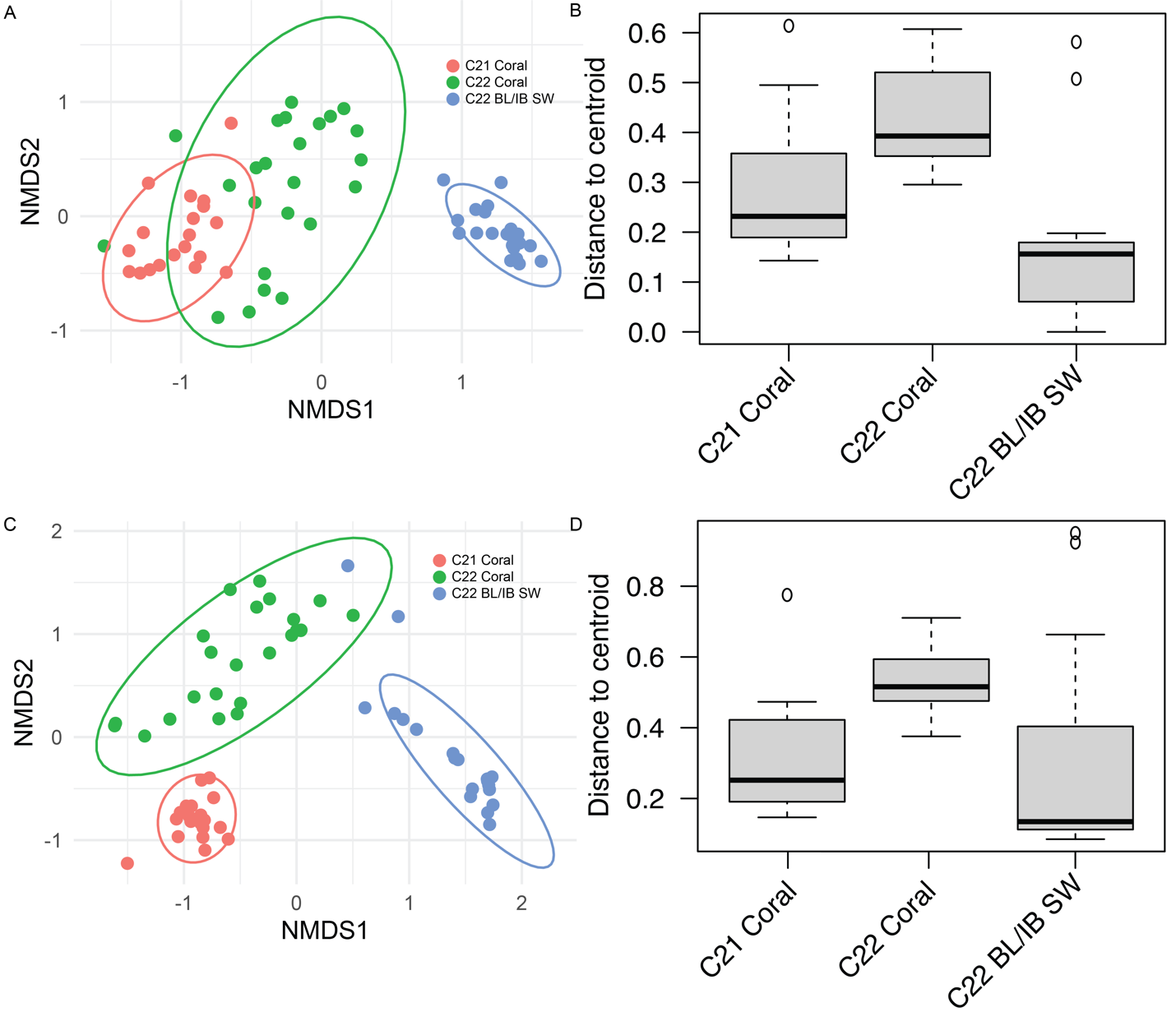
**

**Figure S3 | Non-metric multidimensional scaling (NMDS) and beta dispersion of bacterial and viral communities by sample collection year and sample type.** (A) NMDS plot of bacterial communities in Curaçao 2021 corals (C21 Coral), Curaçao 2022 corals (C22 Coral), and Curaçao 2022 boundary layer (BL) and interbranch (IB) seawater samples (BL/IB SW). Circles denote a 95% CI for the clustered points. (B) Homogeneity of multivariate dispersion evaluated on the Bray-Curtis distance matrices of bacterial communities. (C) NMDS plot of viral communities in Curaçao 2021 corals, Curaçao 2022 corals, and Curaçao 2022 boundary layer and interbranch seawater samples. Circles denote a 95% CI for the clustered points. (D) Homogeneity of multivariate dispersion evaluated on the Bray-Curtis distance matrices of viral communities.

**
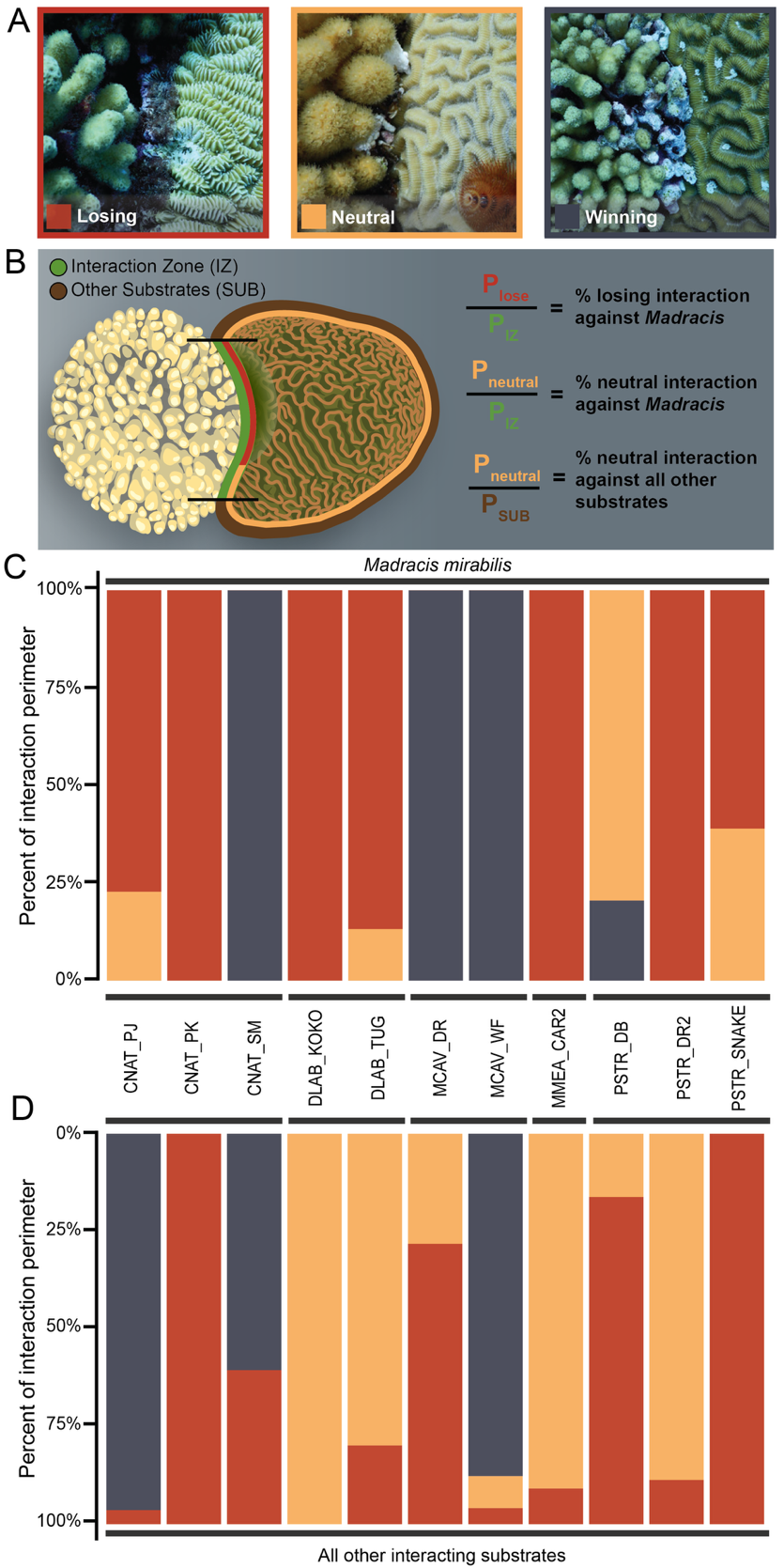
Figure S4 | Interaction outcomes of stony coral species with *Madracis*.** (A) Outlines of the 2D perimeters from high-resolution images were used to calculate the interaction zone perimeter (i.e. perimeter with live *Madracis*; P_IZ_) and the perimeter with all other substrates (P_SUB_). Within each of these perimeters, subsections were characterized as winning, losing, or neutral to calculate the interaction outcomes. Here, we highlight an example where the interacting coral is losing along most of its interaction with live *Madracis* (P_IZ_) and neutral along its entire interaction with other substrates (P_SUB_). (B) Interaction outcomes were described as “losing”, where *Madracis* or other substrates are overgrowing or causing visible damage to the interacting coral; “neutral”, where neither coral nor other substrates are overgrowing or damaging one another; or “winning” where the interacting coral is overgrowing or causing visible damage to the neighboring *Madracis* or all other substrates. (C) The entire perimeter of the interacting coral species (CNAT, DLAB, MCAV, MMEA, and PSTR) was characterized as winning, losing, or neutral against live *Madracis* coral (P_IZ_; top) or (D) all other substrates along its perimeter (P_SUB_; bottom), including, but not limited to coral rubble, turf algae, and sand. Coral species names are abbreviated as follows: *Colpophyllia natans* (CNAT), *Diploria labyrinthiformis* (DLAB), *Montastraea cavernosa* (MCAV), *Meandrina meandrites* (MMEA), and *Pseudodiploria strigosa* (PSTR).

**
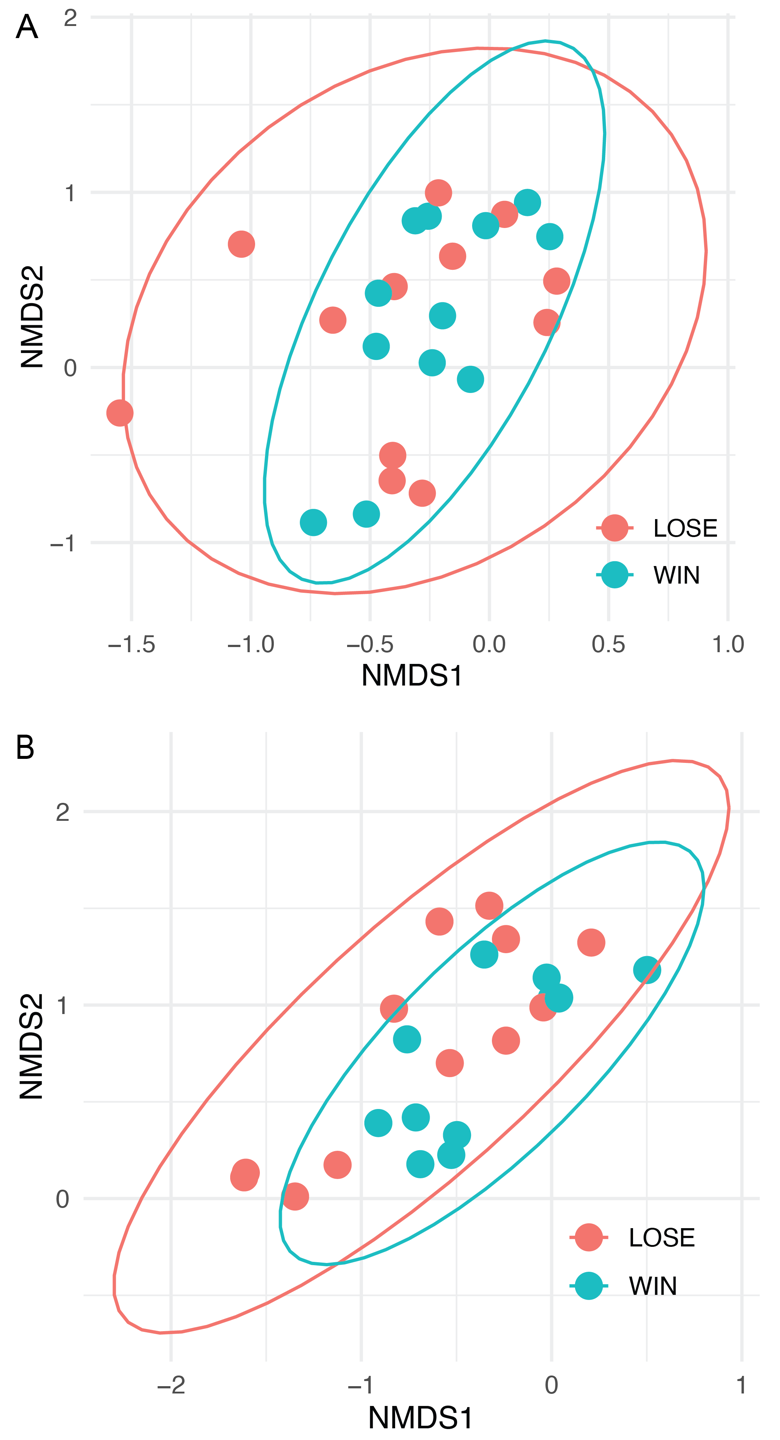
**

**Figure S5 | Non-metric multidimensional scaling (NMDS) of winning vs. losing corals.** (A) NMDS plot of bacterial communities. (B) NMDS plot of viral communities. Circles denote a 95% confidence interval for each sample type.


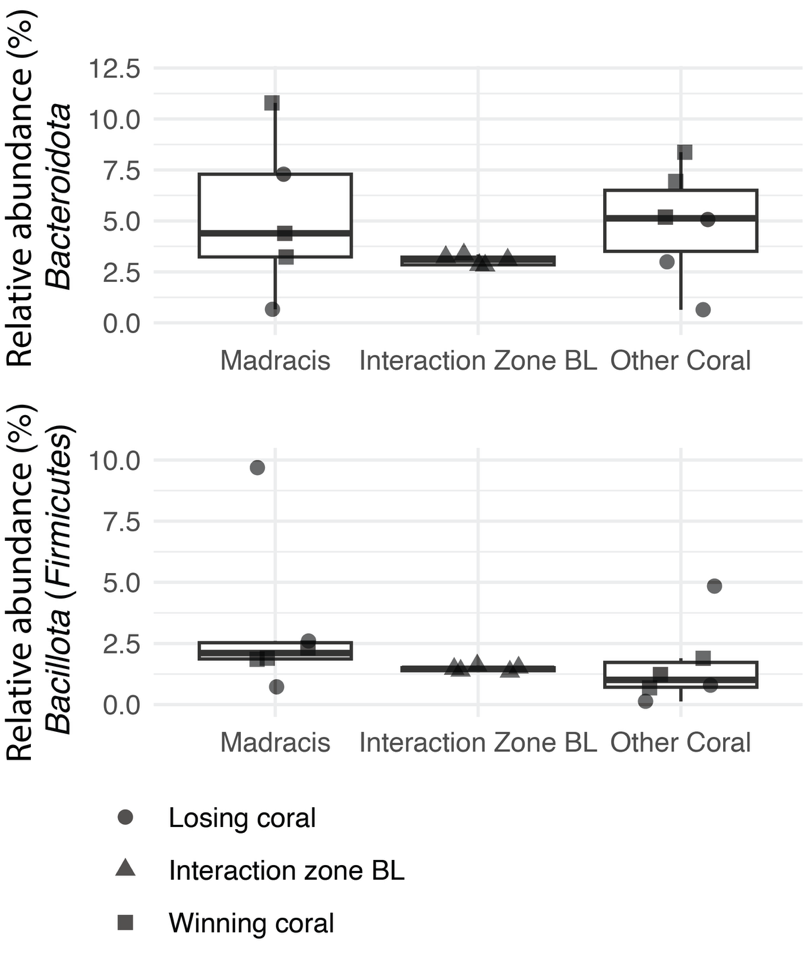


**Figure S6 | Box plots indicating the percent relative abundance of *Bacteroidetes* and *Bacillota* (previously *Firmicutes*).** Bacterial phyla used to predict dysbiosis and coral-turf algae interaction outcomes in previous work were unable to predict interaction outcomes in this study. The x-axis represents metagenomic samples relevant to the interaction (*Madracis* at the interaction zone (CRL3), interaction zone boundary layer seawater (BL2) the interacting coral at the interaction zone (CRL5). The y-axis represents the relative abundance of *Bacteroidetes* (top) and *Bacillota* (bottom) in samples of each type. Circles represent individual samples of losing corals, triangles represent individual samples of interaction zone BL seawater, and squares represent individual samples of winning corals.

**
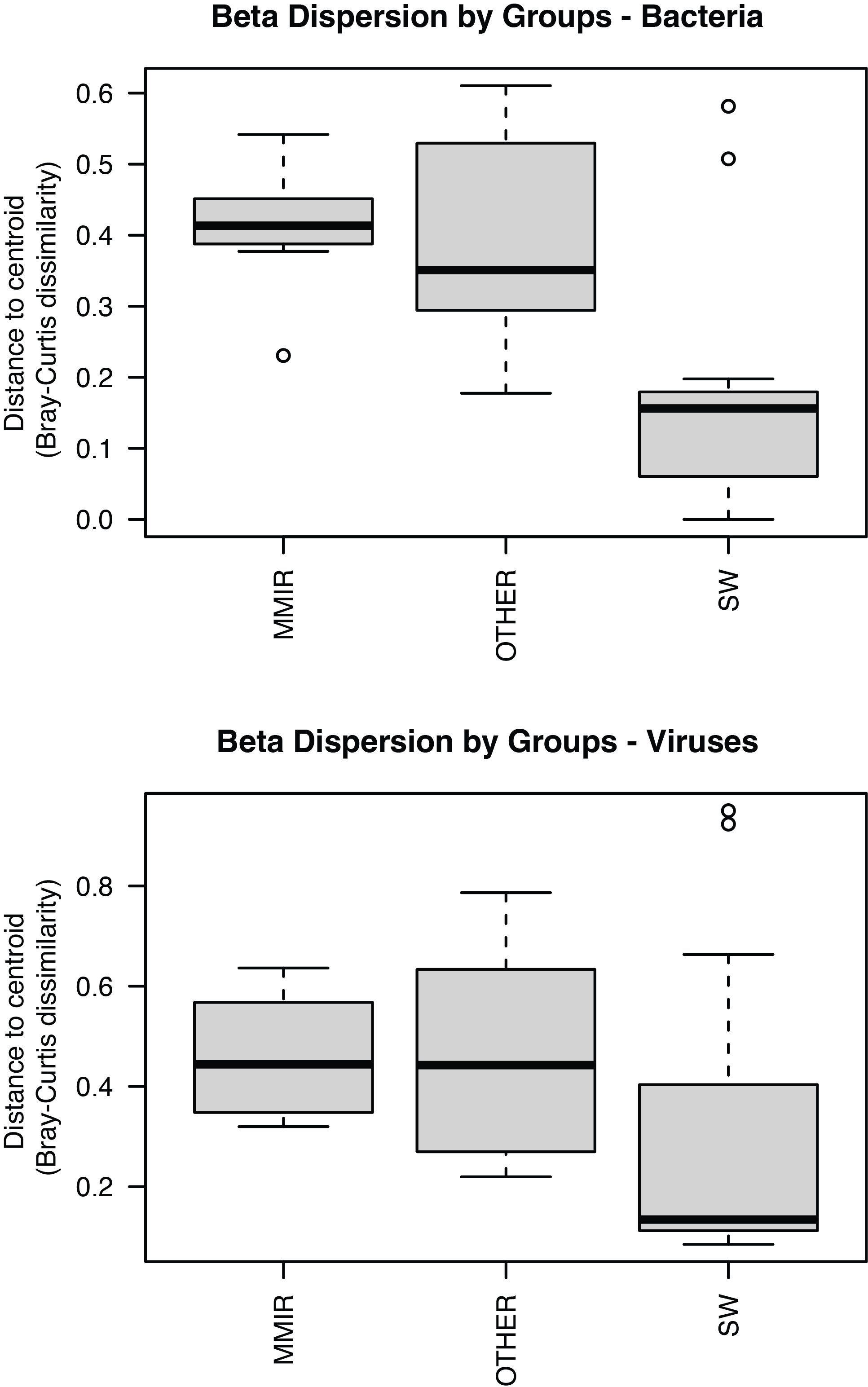
**

**Figure S7 | Beta dispersion of bacterial and viral communities.** Homogeneity of multivariate dispersion was evaluated on the Bray-Curtis distance matrices, where dissimilarity of communities is represented as a unitless value from 0 (identical composition) to 1 (no overlap). In bacterial communities (top), beta dispersion was significantly different between CBL seawater and *Madracis* (MMIR; p = 0.000002) and between CBL seawater and other coral species (p = 0.000000). In viral communities (bottom), beta-dispersion was significantly different between CBL seawater and other coral species (p = 0.0113126).

**
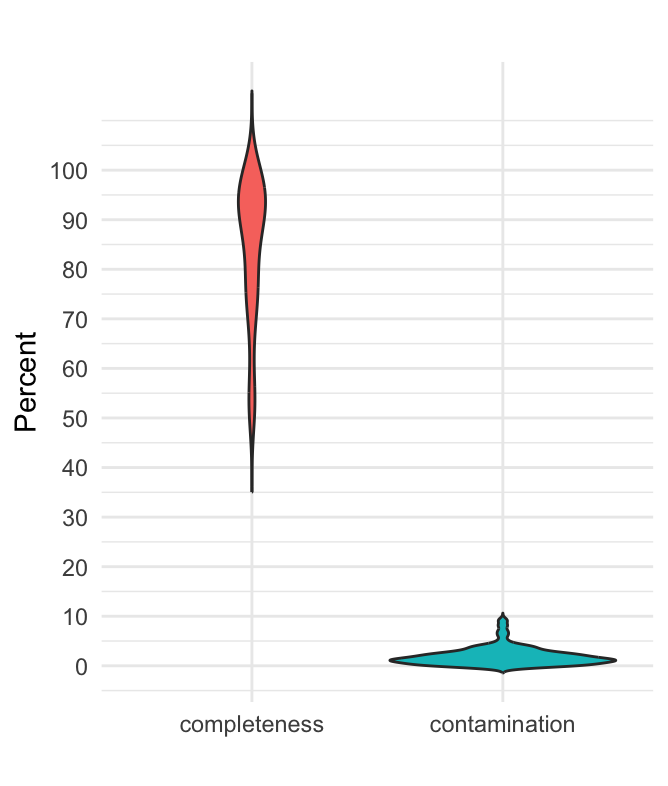
**

**Figure S8 | Completeness and contamination of representative bacterial metagenome-assembled genomes.** Violin plot depicting the completeness and contamination of the representative bMAGs (dereplicated with a 95% similarity threshold). Completeness ranged from 51.03% to 100% (83.90%±1.62%; mean, SE), and contamination ranged from 0.00% to 9.19% (1.91%±0.20%; mean, SE), among the 77 genomes.

**
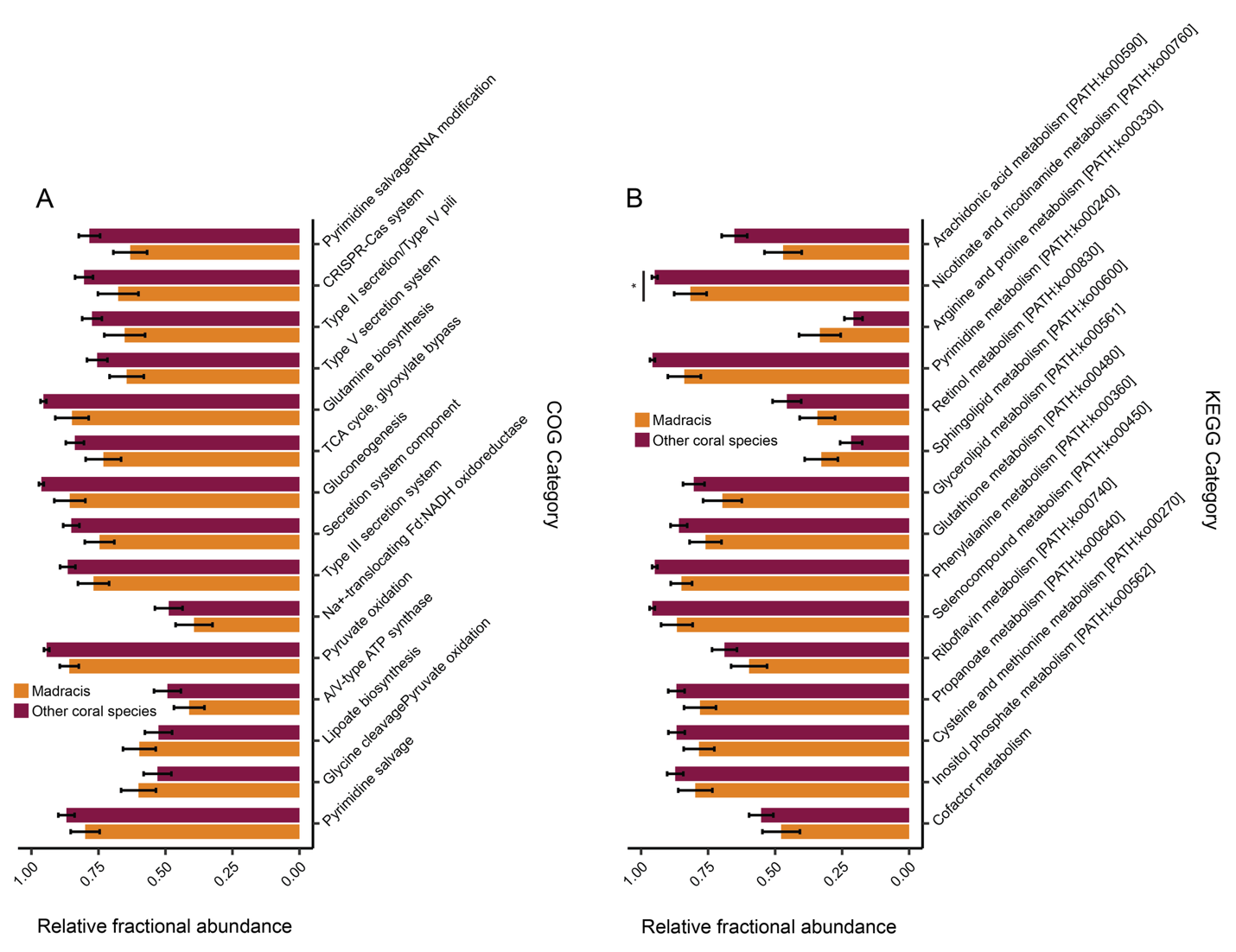
**

**Figure S9 | Differentially abundant COG and KEGG metabolism pathways identified in bacterial metagenome assembled genomes (bMAGs).** The top 15 most differentially abundant pathways between *Madracis* and other coral species from most (top) to least (bottom) differentially abundant. (A) COG pathways and (B) KEGG metabolism pathways were identified by MetaCerberus v1.4.0. P-values shown were adjusted for multiple comparisons using the Benjamini-Hochberg method (FDR control; Significance codes: 0 ‘***’, 0.001 ‘**’, 0.01 ‘*’).


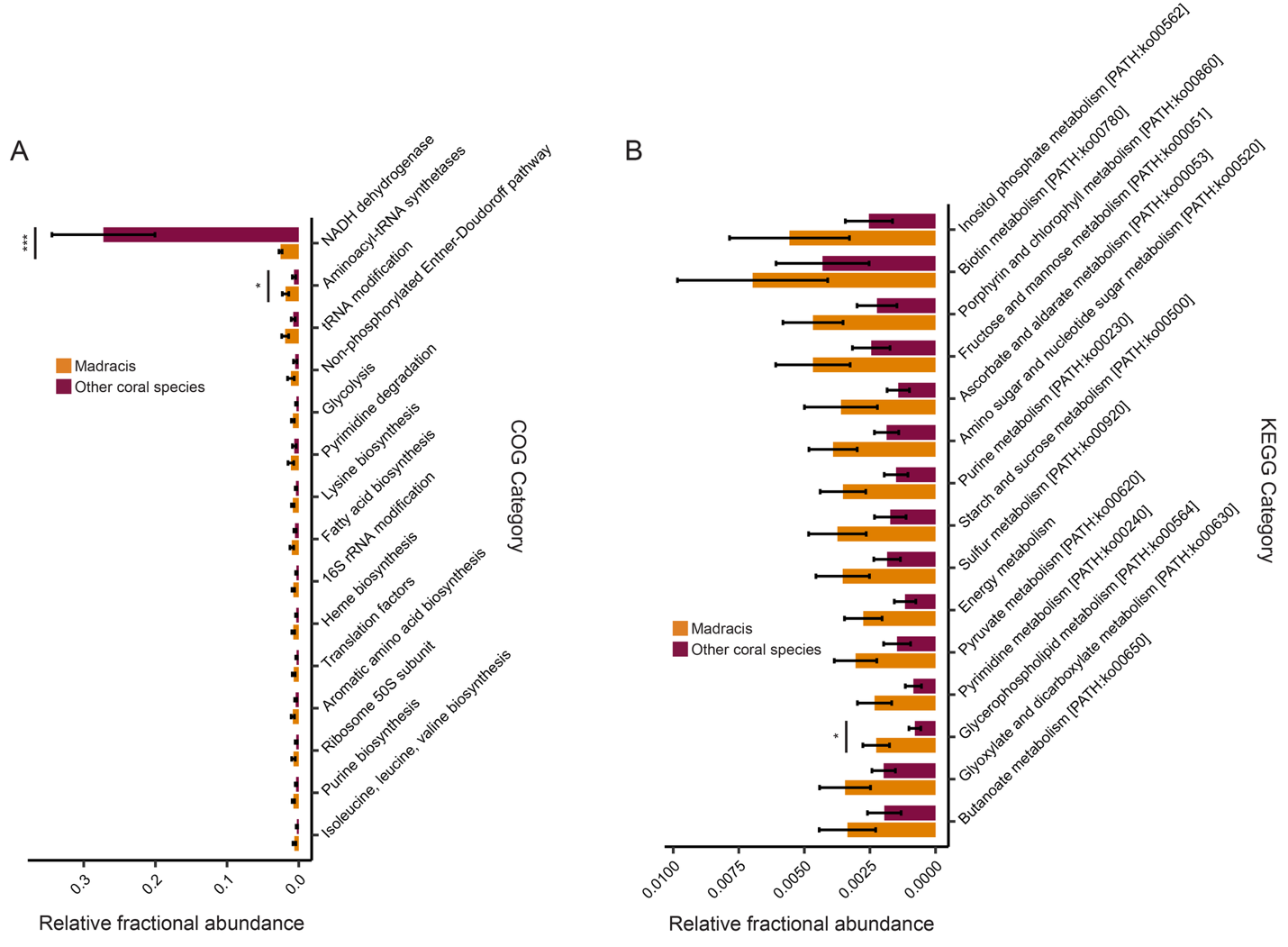


**Figure S10 | Differentially abundant COG and KEGG metabolism pathways identified in bacterial contigs.** The top 15 most differentially abundant pathways between *Madracis* and other coral species from most (top) to least (bottom) differentially abundant. (A) COG pathways and (B) KEGG metabolism pathways were identified by MetaCerberus v1.4.0. P-values shown were adjusted for multiple comparisons using the Benjamini-Hochberg method (FDR control; Significance codes: 0 ‘***’, 0.001 ‘**’, 0.01 ‘*’).

**
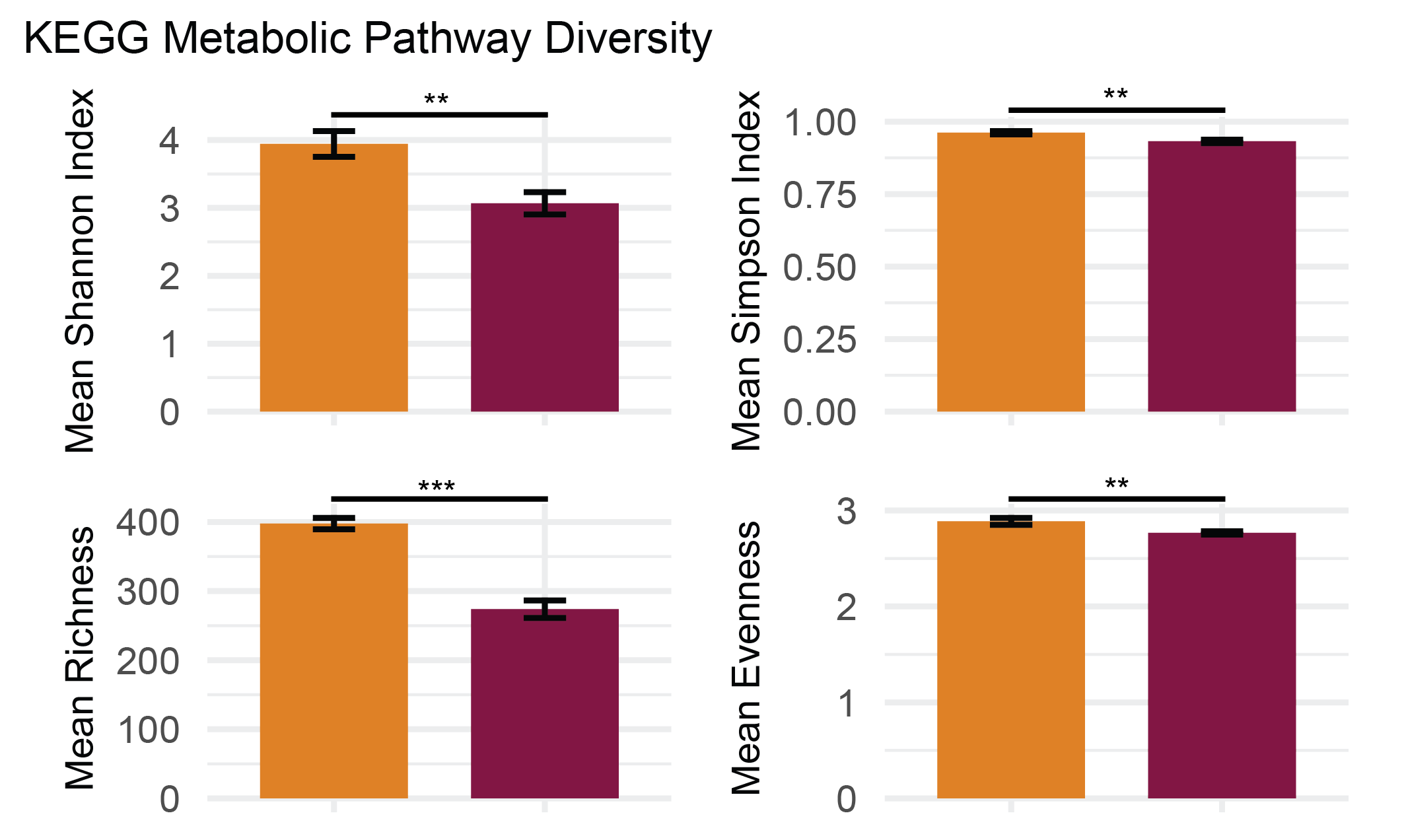
**

**Figure S11 | Diversity of metabolic pathways among bacterial contigs (bMAGs).** (A) Shannon diversity, (B) Simpson diversity, (C) richness, and (D) evenness of KEGG metabolic pathways, showing alpha diversity metrics across the two coral groups. All metrics are based on functional genes identified in bacterial contigs >2,500 bp. These results highlight differences in the metabolic diversity between Madracis and other coral species (Significance codes: 0 ‘***’, 0.001 ‘**’, 0.01 ‘*’).

**
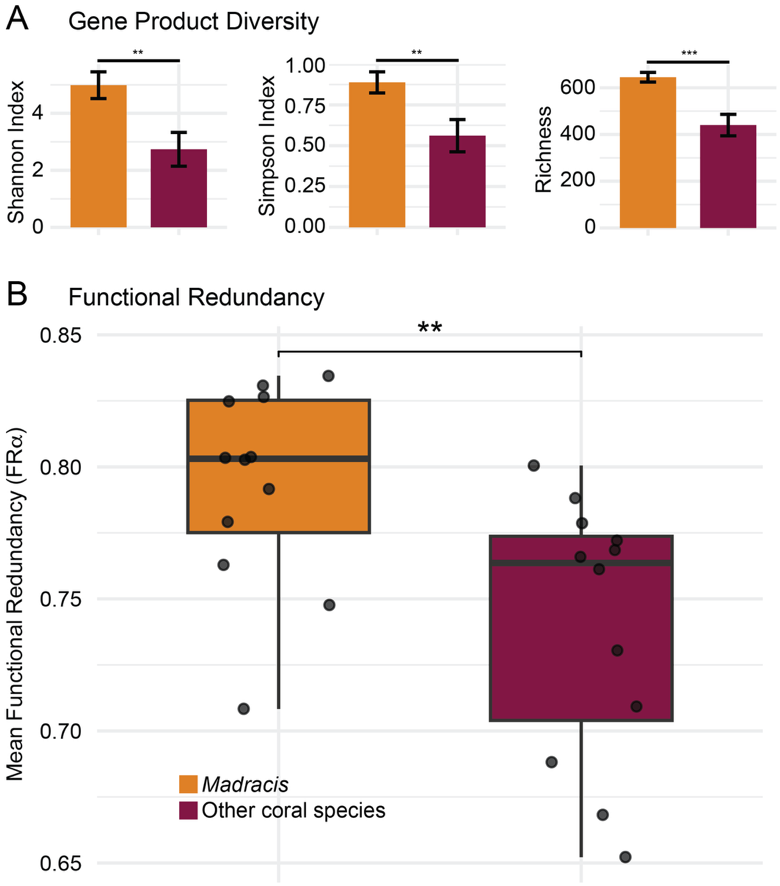
**

**Figure S12 | Functional gene diversity and redundancy in bacterial communities associated with *Madracis* and other coral species.** (A) Shannon diversity, (B) Simpson diversity, and (C) observed richness of functional genes, showing alpha diversity metrics across the two coral groups. (D) Functional redundancy of the bacterial communities, representing the average number of genes contributing to each function. All metrics are based on functional genes identified in bacterial contigs >2,500 bp that were identified at the genus level. Together, these results highlight differences in both diversity and functional buffering capacity between Madracis and other coral species (Significance codes: 0 ‘***’, 0.001 ‘**’, 0.01 ‘*’).

**Supplementary Table Captions:**

*** all tables are uploaded as .xlsx files ***

**Table S1 | Curaçao 2022 coral and coral boundary layer (CBL) seawater sample metadata.** Metadata for the coral- and CBL seawater-associated metagenomes and microscopy samples collected in 2022. These metadata include the sample ID, year, sample type (metagenome vs. microscopy), sample collection date, affiliation, consent, sample description, coral host species, depth of sampling, as well as the country, site, and GPS coordinates of sample collection. For sequence data, the sequencing instrument (if applicable), BioProject, BioSample, accession number, sequencing depth, and total mapped reads (bacterial and viral) are also included.

**Table S2 | Statistical comparisons of NMDS ordinations by sampling year (2021 vs. 2022) and sample type (coral vs. CBL seawater).** Permanova analysis, Tukey HSD pairwise comparisons, and beta dispersion were compared between the three groups. Non-significant values are colored in red.

**Table S3** | **Bacterial community pairwise Adonis p-values.** Permutational multivariate analysis of variance (PERMANOVA) on the Bray-Curtis distance matrix of bacterial relative abundances indicates significant and non-significant differences in bacterial community composition. Non-significant values are colored in red.

**Table S4** | **Viral community pairwise Adonis p-values.** Permutational multivariate analysis of variance (PERMANOVA) on the Bray-Curtis distance matrix of viral relative fractional abundances indicates significant and non-significant differences in viral community composition. Non-significant values are colored in red.

**Table S5 | Bacterial community pairwise Adonis p-values with Curaçao 2021 (OFAV) samples removed.** Permutational multivariate analysis of variance (PERMANOVA) was performed on the Bray-Curtis distance matrix of bacterial relative abundances after removing all 2021 *Orbicella faveolata* (OFAV) samples. Table indicates significant and non-significant differences in bacterial community composition. Non-significant values are colored in red.

**Table S6 | Viral community pairwise Adonis p-values with Curaçao 2021 (OFAV) samples removed.** Permutational multivariate analysis of variance (PERMANOVA) was performed on the Bray-Curtis distance matrix of viral relative fractional abundances after removing all 2021 *Orbicella faveolata* (OFAV) samples. Table indicates significant and non-significant differences in viral community composition. Non-significant values are colored in red.

**Table S7** | **Tukey HSD p-values of mean diversity metrics in groups (*Madracis*, other corals, and CBL seawater).** Here the values are reported for all samples (ALL) and for values calculated with the “failed” coral samples removed (Δ Failed Samples). “Failed” coral samples are those where <1M quality-controlled reads were generated. When these “failed” samples are removed from the bacterial community analysis, Shannon Diversity becomes significantly different between *Madracis* and other coral species, and the difference between the mean richness of these groups is no longer statistically significant. Among the viral community, all trends in significance remain when the failed samples are removed, except for the evenness, which only becomes significantly different between groups after the “failed” samples are removed.

**Table S8** | **T-tests comparing the mean relative abundance of proviruses across the two coral groups.** Rows annotated with “(Δ Failed Samples)” include the same tests with the coral samples that had < 1M reads removed. Rows annotated with “(Δ C21 Corals)” include the same tests with the Curaçao 2021 *Orbicella faveolata* samples removed. Columns MMIR, OTHER, and CBL SW indicate the mean relative abundance of proviruses in each group as a decimal fraction.

**Table S9 | Coral boundary layer (CBL) microscopy counts by sample type.** The mean and standard error (SE) are reported for VLP per mL, Cells per mL, and VMR across each of the four sample types.

**Table S10 | Statistical comparisons of interbranch and boundary layer microscopy data.** Table displays the p-values resulting from Tukey HSD pairwise comparisons of virus-like particles (VLP) per mL, cells per mL and virus-to-microbe ratios between interbranch (IB) and boundary layer (BL) water samples. Non-significant values are colored in red.

**Table S11** | **Bacterial and viral community pairwise Adonis p-values in coral boundary layer samples.** Permutational multivariate analysis of variance (PERMANOVA) on the Bray-Curtis distance matrix of bacterial and relative abundances and viral relative fractional abundances indicates significant and non-significant differences in bacterial community composition. Non-significant values are colored in red.

**Table S12 | Bacterial indicator species of losing coral interactions.** Indicator species analysis was performed using the multipatt function in the R package indicspecies. This function uses a multi-level pattern analysis that calculates an indicator value for each group. Here we display all statistically significant indicator bacterial genera. This revealed 7 bacterial genera that were primarily found in corals that were losing and were present across most losing samples.

**Table S13 | Top 20 bacterial genera contributing to the dissimilarity between *Madracis* and other corals.** Similarity percentage (SIMPER) analysis showing the bacterial genus, average contribution to dissimilarity, standard deviation (SD), ratio (average/SD), the average abundance in *Madracis*, the average abundance in other coral species, and the relative abundance (where “+” indicates higher average abundance in *Madracis*, relative to other coral species).

**Table S14 |** **Taxonomy of bacterial metagenome-assembled genomes (bMAGs).** Taxonomic classification of bacterial metagenome-assembled genomes (bMAGs). The table presents the taxonomic assignment of bMAGs, including bin ID, domain, phylum, class, order, family, genus, and species, as determined by GTDB-Tk v2.4.0 (GTDB release 220).

**Table S15 | Top 20 viral genomes and genome fragments contributing to the dissimilarity between *Madracis* and other corals.** Similarity percentage (SIMPER) analysis showing the viral genome or genome fragment, average contribution to dissimilarity, standard deviation (SD), ratio (average/SD), the average abundance in *Madracis*, the average abundance in other coral species, the relative abundance (where “+” indicates higher average abundance in *Madracis*, relative to other coral species), and the iPHoP predicted host.

**Table S16 | Viral indicator species in *Madracis* metagenomes.** Indicator species analysis was performed using the multipatt function in the R package indicspecies. This function uses a multi-level pattern analysis that calculates an indicator value for each group. Here we display the indicator viruses with ≥ 95% specificity and fidelity. This revealed 44 viruses that are both primarily or exclusively found in *Madracis* samples and found across most or all *Madracis* samples. Table includes iPHoP predicted hosts.
